# Elevated dNTP Pool Levels Impair Homologous Recombination and Enhance Glioblastoma Sensitivity to Irradiation and Temozolomide

**DOI:** 10.3390/ijms27188045

**Published:** 2026-09-10

**Authors:** Dominique Monroe, Mercy Kehinde-Ige, Arilyn Williams, Vafa Ismayilova, Apeksha Anand, Matthew Kededa, Ramsha Khanam, Aman Kalsi, Ali S. Arbab, Daitoku Sakamuro, Huidong Shi, Waaqo Daddacha

**Affiliations:** 1 Department of Biochemistry and Molecular Biology, Medical College of Georgia, Augusta University, Augusta, GA 30912, USA; dominique.monroe@vai.org (D.M.); mkehindeige@augusta.edu (M.K.-I.); awilliams33@augusta.edu (A.W.); vismayilova@augusta.edu (V.I.); mkededa@augusta.edu (M.K.); rkhanam@augusta.edu (R.K.); akalsi@augusta.edu (A.K.); aarbab@augusta.edu (A.S.A.); dsakamuro@augusta.edu (D.S.); hshi@augusta.edu (H.S.); 2 Georgia Cancer Center, Augusta University, Augusta, GA 30912, USA; apanand@augusta.edu; 3 Immunology Center of Georgia, Augusta University, Augusta, GA 30912, USA

**Keywords:** Glioblastoma, homologous recombination, DNA damage, double-strand break, double-strand break repair, DNA end resection, polymerase α, dNTP pool

## Abstract

Glioblastoma (GBM) standard of care includes surgical resection followed by ionizing radiation (IR) and Temozolomide, which induce DNA double-strand breaks. Homologous recombination (HR), a critical DNA double-strand break repair pathway, is augmented in GBM, contributing to resistance and poor patient outcomes. Here, we demonstrate that increasing deoxyribonucleoside triphosphate (dNTP) levels impairs HR-mediated double-strand break repair, rendering GBM cells sensitive to IR and Temozolomide. Interestingly, combining an elevated dNTP pool level with IR and/or Temozolomide promotes the recruitment of DNA polymerase-α/primase, which is typically involved in Okazaki fragment synthesis during DNA replication, to the DNA double-strand break site, thereby interfering with DNA end resection. Specifically, higher dNTP pool levels disrupted the recruitment of HR-associated proteins such as RPA70 and RAD51, an effect reversed by Aphidicolin, a DNA polymerase-α/primase inhibitor. Impaired HR delayed IR- and/or Temozolomide-induced DNA double-strand break repair, leading to growth arrest and apoptosis. Furthermore, higher dNTP pool levels led to downregulation of DNA replication and HR-associated genes, while upregulating several pro-apoptotic genes. Increased sensitivity to IR and Temozolomide was also observed in engineered IR-resistant GBM cell lines and in naturally recurrent patient-derived GBM cells that emerge post-therapy. These findings emphasize how dNTP pool levels regulate HR and uncover a promising vulnerability that could be exploited to overcome resistance to DNA-damaging treatments in GBM and beyond.

## 1. Introduction

Glioblastoma (GBM) is a deadly primary brain tumor. GBM standard of care constitutes ionizing radiation (IR) and genotoxic chemotherapeutic agents such as Temozolomide (TMZ), which exert their effects by inducing lethal DNA double-strand breaks (DSBs) [1,2]. However, GBM often exhibits resistance to these therapeutics due to dysregulated DNA repair mechanisms, presenting a significant barrier that contributes to poor patient outcomes [3,4]. Targeting rapidly dividing cancer cells with IR and some genotoxic agents results in the accumulation of DSBs, which, if left unrepaired, trigger apoptosis [5]. Despite the aggressive treatment strategy, there has been no significant improvement in the five-year survival rate for GBM patients since TMZ and radiation therapy were established as the standard of care 20 years ago [6,7,8]. Resistance to TMZ is frequently driven by high expression of O6-methylguanine-DNA methyltransferase (MGMT), which facilitates repair of TMZ-induced DNA lesions [6,7,8]. Although several approaches have been explored to target DSB repair proteins, resistance remains a barrier to meaningful progress [9,10], underscoring the need for a novel strategy to disrupt DNA repair in GBM.

Homologous recombination (HR) is an accurate and preferred pathway for DSB repair and is instrumental in cancer cell survival following genotoxic assault [11]. DSBs are repaired through two major pathways, non-homologous end joining (NHEJ) and HR. While NHEJ is active in all cell cycle phases, HR is limited to S and G2 phases, where sister chromatids are available to provide an undamaged template for repair. HR consists of three main phases: presynapsis (DNA end processing), synapsis (homology search and identification), and post-synapsis (strand invasion and resolution) [12]. The first phase involves DNA end resection mediated by several key players, including the MRN complex (MRE11, RAD50, and NBS1), CtIP, EXO1, DNA2, SAMHD1, and BRCA1. This step generates 3′ overhangs ranging from a few hundred bases to multiple kilobases of single-stranded DNA, depending on the cell type and context [13]. Replication Protein A large subunit (RPA70), a single-stranded DNA-binding protein, rapidly binds and protects it from self-annealing and nuclease-mediated degradation [14,15]. Successful end resection allows for transition into homology search and strand invasion. BRCA2 facilitates the replacement of RPA with RAD51, a recombinase essential for HR. RAD51 forms a nucleoprotein filament on the single-stranded DNA to search for and invade the homologous DNA sequence on the sister chromatid [12,16]. The strand invasion step forms a displacement loop (D-loop), which serves as a template for DNA synthesis, leading to resolution [17].

DNA-end resection is necessary for successful HR progression [18]. However, the extensive amount of single-stranded DNA generated during this step introduces potential vulnerabilities. Under suitable conditions, resected single-stranded DNA can become an unintended substrate for DNA polymerases [19,20,21]. DNA polymerase engagement with the resected DNA can be influenced by the cellular deoxyribonucleoside triphosphate (dNTP) levels, which can promote DNA polymerase -mediated resected DNA synthesis. This premature synthesis of resected DNA could interfere with sufficient single-stranded DNA generation and subsequently undermine the homology search process, leading to inefficient repair and increased sensitivity to DSB-inducing agents [13]. Therefore, it is essential to elucidate how variations in the dNTP levels affect HR integrity. However, this possibility has yet to be investigated, and few studies have explored the synthesis of resected DNA ends. Previous studies have demonstrated that reducing the dNTP levels leads to replication stress, cell cycle arrest, and cell death [22]. While cellular dNTPs are necessary for DSB repair, their levels remain unchanged after inducing DSBs [23,24], suggesting that tightly regulated dNTP levels following genotoxic therapy are crucial. These observations led us to investigate how elevated dNTP levels affect DNA repair. Here, we demonstrate that elevated cellular dNTP levels impair HR by promoting engagement of DNA polymerase α/primase 1 (DNA Pol α), leading to the accumulation of DSBs and apoptosis. While elevated dNTP levels increased GBM cell sensitivity to IR and TMZ, the benefit was most pronounced in engineered IR-resistant and patient-derived recurrent GBM cells.

## 2. Results

### 2.1. Elevating Intracellular dNTP Levels Decreases Cell Viability and Promotes Apoptosis Following Ionizing Radiation

**Figure 1:**
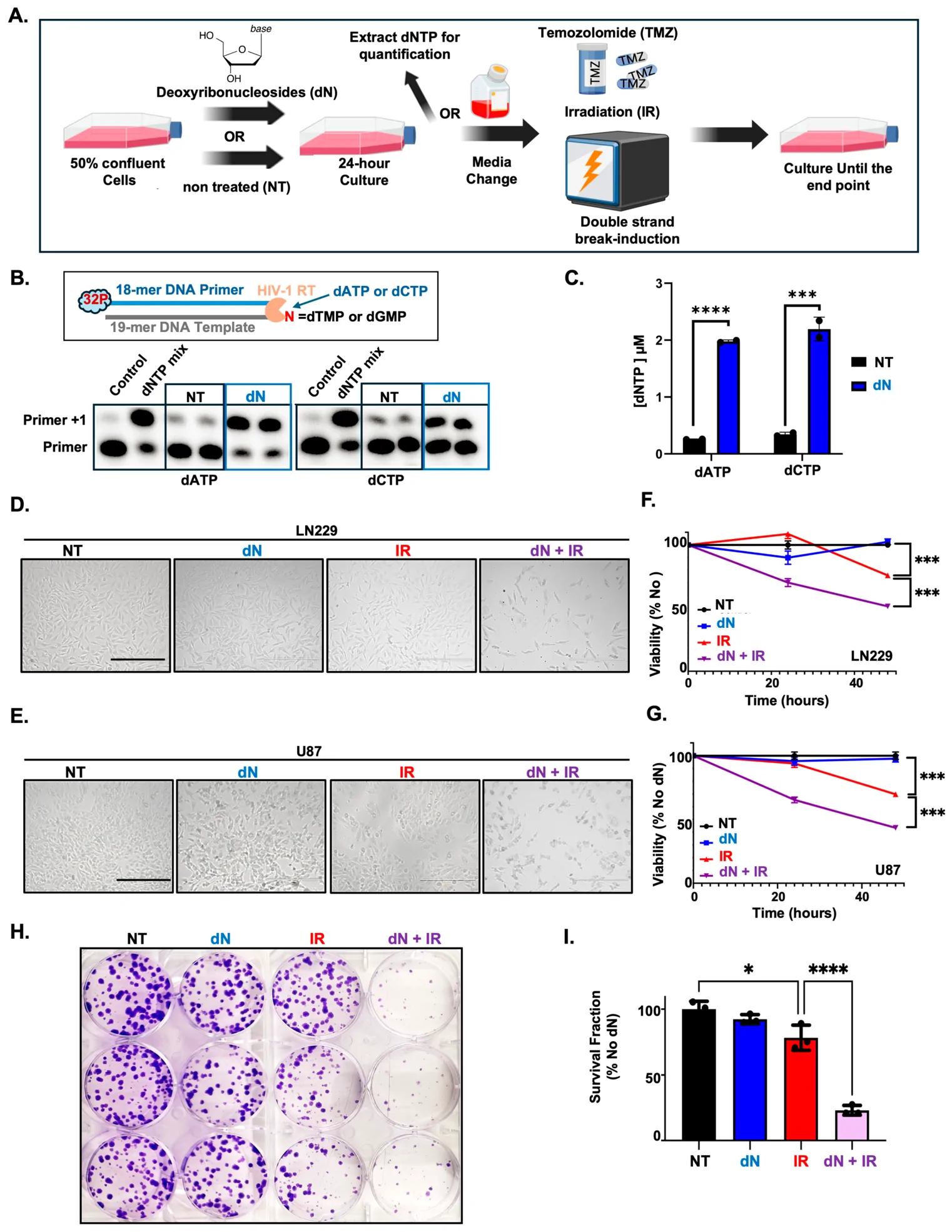
**Elevating intracellular dNTP levels decreases GBM cell viability after IR.** (**A**): Schematics of the experimental procedure for deoxynucleoside (dN) treatment (0.5 mM), deoxyribonucleotide (dNTP) extraction, and exposure to IR (5 Gy) and/or TMZ (200 µM). (**B**,**C**): Primer extension assay was used to quantify the dATP and dCTP levels. 18-mer γ−^32^P-radiolabeled primer was annealed to a 19-mer template, creating a one-nucleotide overhang (N) to accommodate the addition of a single complementary nucleotide. dNTPs extracted from cells under the indicated condition were used as a substrate to extend the 18-mer primer by HIV-1 Reverse transcriptase. In the figure, “Primer” indicates the nonextended 18-mer primer, and Primer + 1 indicates an 18-mer primer with one additional nucleotide. Technical positive control (dNTP mix) and negative control (control) were included to confirm the assay works as intended. (**D**,**E**): Cells were treated with dNs or PBS for 24 h, and images were obtained 48 h later. Bright-field representative images are presented for LN229 in (**D**) and U87 in (**E**). Scale bar: 400 µm. (**F**,**G**): Cells were treated with dN for 24 h, treated as indicated, and viability was assessed. The non-treated (NT) population was used as a reference positive control (100%) for each time point. Graphs presented are for LN229 in F and U87 in (**G**). Scale bar: 400 µm (Two-Way ANOVA). (**H**,**I**): Clonogenic assay of LN229 cells. Cells were treated with dN for 24 h. 300 cells/well were seeded and irradiated. Cells were incubated for 14 days, at which point 50 cells/colony were achieved in the untreated group. (**H**): Representative image. (**I**): Quantification of percent survival fractions 14 days after treatment. The error bars represent biological triplicates (Student’s *t*-test). Control (Water), dNTP mix (0.5 mM dNTP mix), NT (Non-treated), dN (0.5 mM dN mix), IR (5 Gy Irradiation), dN + IR (dN and IR Combination treatment). * *p* < 0.05, *** *p* < 0.001, **** *p* < 0.0001.

The resistance of cancer cells to ionizing radiation remains a significant obstacle in current treatment strategies [3,25]. Given that the cellular dNTP levels are maintained at baseline following exposure to DNA-damaging agents [23], we reasoned that perturbing this balance could increase GBM cell sensitivity to IR and TMZ by undermining recovery capability. To test this, we used LN229 and U87, established GBM cell lines [26], that have been characterized as radio-resistant [27,28,29]. For all experiments, cells were pretreated with deoxynucleosides (dN), precursors that will be phosphorylated to generate dNTPs, exposed to TMZ, IR, or the TMZ and IR combination (TMZ + IR), and allowed to recover (Figure 1A). Supplementation of cells with dN has been used previously to increase the cellular dNTP levels in non-dividing cells, but has not been validated in GBM cells [30]. Therefore, we assessed the feasibility of increasing the dNTP pool in GBM cells using HIV-RT-based dNTP treatment. As shown in Figure 1B,C, where the dNTP levels were quantified by calculating an extended and unextended 32P-labeled primer, we observed a ~4-fold increase in dATP and dCTP levels following dN treatment, providing a valuable tool for assessing the impact of dNTP pool levels on DNA repair and the efficacy of IR and TMZ. The increment was proportional to the concentration of supplemented dN and plateaued at 0.5 mM (Supplementary Figure S1A,B). Interestingly, cells pre-treated with dNs exhibited increased sensitivity to IR, as demonstrated by reduced cell growth or survival in both LN229 (Figure 1D) and U87 (Figure 1E). These findings were supported by significantly reduced cell viability following dN and IR combination treatment in LN229 (Figure 1F) and U87 (Figure 1G). Consistent with these results, dN-treated GBM cells’ colony formation efficiency was substantially decreased 14 days post-IR exposure (Figure 1H,I), directly linking the observed viability to enhanced IR efficiency. Similarly, in U2OS, a cell line widely used for DNA repair analysis, cell growth and viability decreased substantially following dN and IR combined treatment (Supplementary Figure S1A,B). These findings demonstrate that elevating GBM cellular dNTP pool levels potentiates sensitivity to genotoxic agents such as IR.

### 2.2. Elevated dNTP Pool Levels Suppress Expression of Genes Associated with DNA Repair, Replication, and Anti-Apoptosis, and Lead to Apoptosis in Combination with IR or TMZ

**Figure 2:**
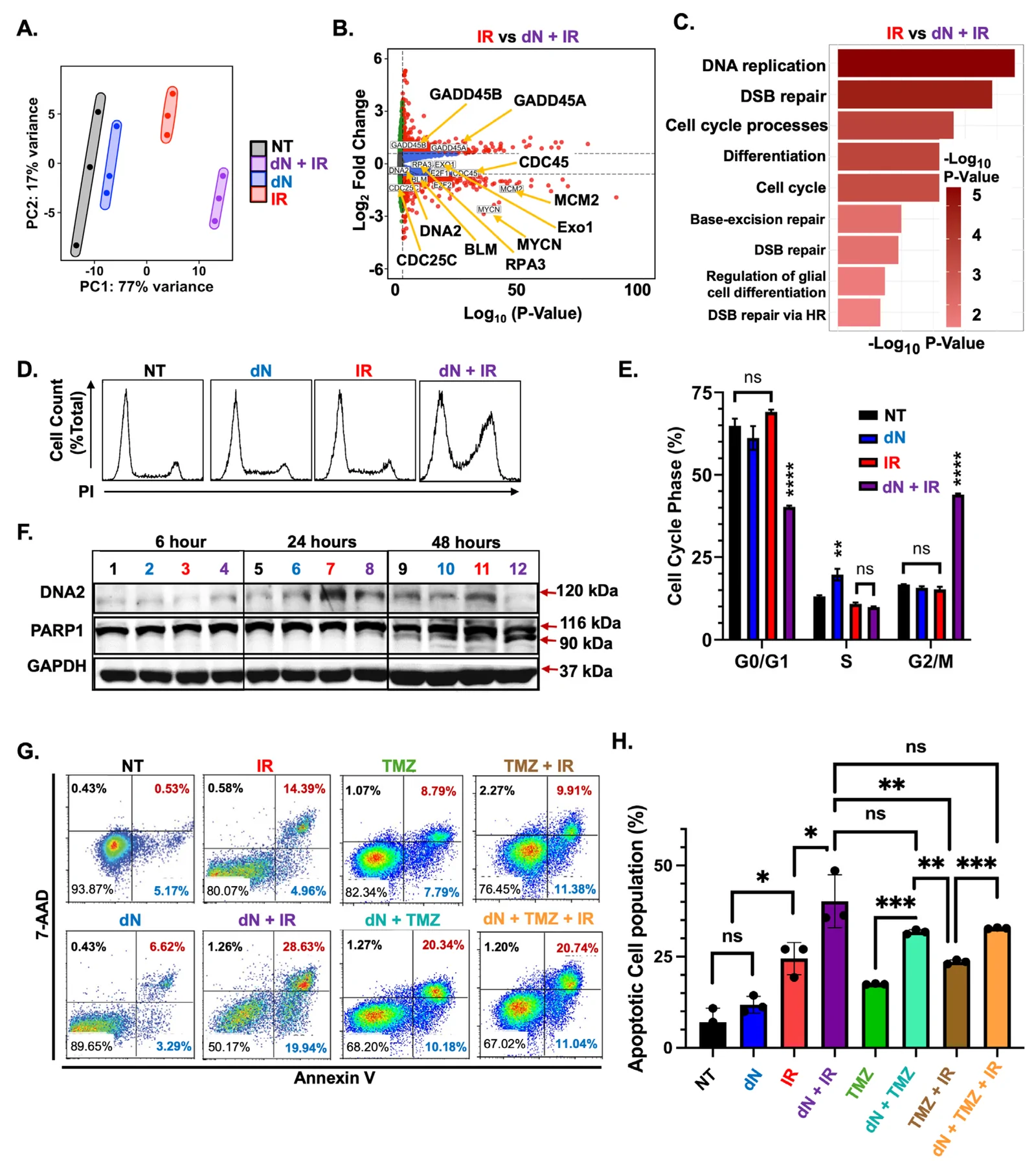
**Transcriptomic alterations in genomic maintenance and stability pathways reveal that genotoxic therapy induces cell cycle arrest and increases apoptosis when dNTP levels are elevated.** (**A**): Principal Component Analysis (PCA) of transcriptome data for LN229 cells treated with PBS (control), 0.5 mM dNs (dN only), 5 Gy IR (IR only), or 0.5 mM dNs + 5 Gy IR (dN + IR). (**B**): Volcano plot showing gene expression changes in IR-only compared with dN + IR-treated groups, highlighting upregulated genes in red and down-regulated genes in blue (*p* < 0.05, and fold change > 1.5). (**C**): Gene ontology enrichment analysis of selected pathways exhibiting significant changes between IR only vs. dN + IR. (**D**): Cell cycle analysis of LN229 cells 48 h after the indicated treatment. (**E**): Quantification of cell cycle phase distribution (Student’s *t*-test). (**F**): Immunoblot of LN229 lysates 6, 24, and 48 h after the indicated treatment. DNA Replication Helicase/Nuclease 2 (DNA2) and Poly(ADP-Ribose) Polymerase 1 (PARP1) were probed. Glyceraldehyde-3-phosphate dehydrogenase (GAPDH) was used as a loading control. Numbers represent: lanes 1,5, and 9 = no treatment; lanes 2, 6, and 10 = dN alone; lanes 3, 7, and 11 = IR alone; and lanes 4, 8, and 12 = dN + IR combination treatment. (**G**): Annexin V/7AAD staining of LN229 cells 48 h after the indicated treatment to determine early (Quadrant 4-Blue) and late-stage apoptosis (Quadrant 2-Red) and necrosis (Quadrant 1-Black). (**H**): Quantification of apoptotic LN229 cells (One-Way ANOVA). NT (non-treated), dN (0.5 mM dN mix), IR (5 Gy Irradiation), dN + IR (dN and IR Combination treatment). ns = non-significant, * *p* < 0.05, ** *p* < 0.01, *** *p* < 0.001, **** *p* < 0.0001.

Genotoxic agents impose stress on cells by eliciting both transcriptional and post-translational responses to DNA damage. To determine whether elevating dNTP pool levels before DSB induction alters gene expression, we performed bulk RNA sequencing at 48 h after IR exposure, with and without dN treatment. Modulation of dNTP levels could affect the mutation rate and undermine our analysis. Therefore, we assessed this possibility following dN treatment. As shown in Supplementary Figure S2A, there was no significant difference in mutation when the dNTP levels were elevated, confirming that dN treatment-derived mutations alone may not contribute to a significant change in gene expression. In the absence of DNA damage, a comparative analysis between the dN-only and untreated groups revealed no significant changes in the pathways associated with apoptosis, DNA repair, or other stress-related pathways (Supplementary Figure S2B,C). Overall, only 84 genes showed statistically significant differential expression when comparing the untreated to dN-treated group. Interestingly, the principal component analysis of transcriptomic data revealed high variability in gene expression between the IR-only and dN + IR combination treatment group (Figure 2A), and we identified 1106 differentially expressed genes (fold-change ≥ 1.5, adjusted *p* < 0.05). Specifically, 344 genes were significantly upregulated, while 762 genes were significantly downregulated (Figure 2B and Supplementary Figure S2D–F). Gene Ontology (GO) Enrichment Analysis revealed 201 significantly altered pathways, with DNA replication, DNA repair, and cell cycle processes being amongst the most affected (Figure 2C and Supplementary Figure S2F,G).

For instance, the expression of critical replication licensing factors such as *CDT-1* and *BLM* was significantly reduced along the *CDC45/MCM* complex, suggesting impaired initiation and elongation phases of DNA replication [31,32]. Simultaneously, *CDC25C* was downregulated, potentially hindering the formation of the cyclin B1/CDK1 complex and leading to G2/M cell cycle arrest [33]. This was supported by increased expression of genes negatively regulating cell cycle progression, as shown in Figure 2B,C and Supplementary Figure S3A. p53-mediated repression of other mitosis-promoting genes provides a critical window for DNA damage assessment and repairing DNA damage [34]. As G2/M arrest persists, cells activate pathways that drive the decision to commit apoptosis. Notably, there was upregulation of *GADD45*, a protein overexpressed during prolonged DNA damage to prevent DNA replication stress [35] (Figure 2B and Supplementary Figure S3B). However, when repair mechanisms are insufficiently engaged, this protective signal is accompanied by a marked upregulation of pro-apoptotic mediators such as *CHOP*, along with a substantial downregulation of the anti-apoptotic factor *BCL-2* [36]. This is consistent with our observation (Figure 2B,C, Supplementary Figure S3B). We also observed the upregulation of *IP3R*, which promotes autophagy and primes cells for programmed cell death [37,38]. In addition to its role in cell cycle arrest, *GADD45* has been associated with apoptosis [39], consistent with the observed upregulation following dN + IR combination treatment (Figure 2B). Furthermore, we observed a significant reduction in several DNA repair and replication genes, including *DNA2*, *EXO1*, *XRCC3*, *RAD54*, *BLM*, *RPA*, *RPA3*, *DNA* ligases, and various components of MCM helicase complexes (Figure 2B**,** Supplementary Figure S3C,D). This shows an overwhelmed DNA repair and replication capacity, which promotes apoptosis [40,41].

In agreement with the observed downregulation of genes associated with cell cycle progression, cell cycle analysis revealed significant G2/M arrest following dN + IR combination treatment (Figure 2D,E). Interestingly, treatment with dN or IR alone did not significantly affect overall cell cycle profiles. (Figure 2D,E, Supplementary Figure S3E). Immunoblotting confirmed the induction of apoptosis, as evidenced by the increased PARP1 cleavage 48 h post dN + IR combination treatment. Additionally, DNA2 and EXO1, critical HR associated proteins, were substantially reduced, paralleling their mRNA downregulation and indicating compromised HR function under elevated dNTP conditions (Figure 2F, Supplementary Figure S3F). We further validated the transcriptomic findings and assessed whether the reduced proliferation and viability observed in Figure 1 were attributable to increased apoptosis following dN + IR combination treatment, using Annexin V and 7AAD staining. TMZ, a standard-of-care regimen for GBM, was included in this assessment. As shown in Figure 2G,H, a significant increase in apoptotic cell populations was observed following dN + IR or dN + TMZ combination treatment as compared with IR or TMZ alone. Notably, a similar apoptosis rate was observed following dN + IR + TMZ triple combination treatment, indicating that elevated dNTP levels sensitize cells even under clinically relevant combination therapy. Although IR, TMZ, or IR + TMZ showed a notable effect, combining them with dN significantly increased apoptosis. Interestingly, combining dN with IR or TMZ yields a greater effect than IR + TMZ, demonstrating the enhanced benefit of elevated dNTP pool levels compared with the currently available standard-of-care combination treatment. dN + IR showed a comparable effect to dN + IR + TMZ, suggesting that a triple treatment could be avoided. Next, we examined whether the increased apoptosis was specifically associated with DSB repair rather than with a generalized increase in apoptotic susceptibility due to elevated dNTP pool levels. Cells treated with staurosporine, a potent apoptosis inducer that acts independently of DNA damage, showed no significant difference in apoptosis following dN treatment (Supplementary Figure S3G,H). This strongly suggests that the elevated apoptosis observed following dN supplementation and genotoxic therapies is linked to impaired DNA repair. These findings show that elevated dNTP pool levels overwhelm DNA repair capacity, shifting the cellular response from an attempted recovery to apoptosis, and demonstrate the benefit of elevated dNTP pool levels when combined with IR, TMZ, or IR + TMZ treatments.

### 2.3. DNA Double-Strand Breaks Persist Longer Under Elevated dNTP Levels

**Figure 3:**
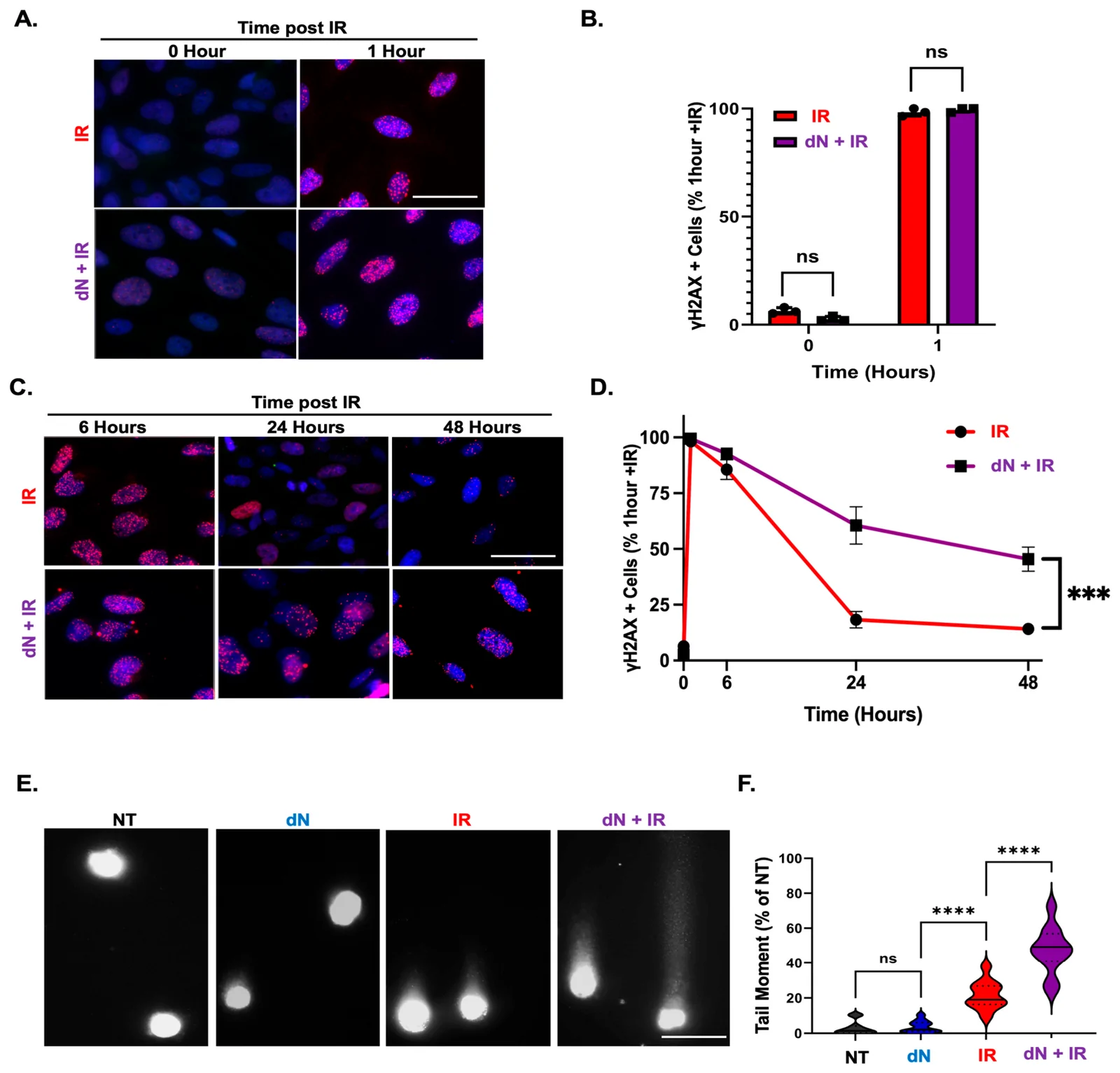
**Elevated dNTP levels delay the repair of DNA double-strand breaks.** (**A**): DAPI (blue) and γH2AX (red) merged representative image before or 1 h after PBS or dN treatment and IR in LN229. Scale bar, 50 µm. (**B**): Quantification of 0-h and 1-h γH2AX foci with the indicated treatment (Student’s *t*-test). (**C**): DAPI (blue) and γH2AX (red) merged representative image of foci at 6-h, 24-h, and 48-h time points of irradiated LN229 cells, with or without dN treatment. Scale bar, 50 µm. (**D**): Quantification of 0-, 1-, 6-, 24-, and 48-h γH2AX foci with the indicated treatment. (**E**): Neutral comet representative images from LN229 cells, dN-treated or untreated cells, with and without exposure to 5 Gy IR after 24 h. Scale bar, 50 µm. (**F**): Quantification of tail moments (One-Way ANOVA). NT (non-treated), dN (0.5 mM dN mix), IR (5 Gy irradiation), dN + IR (dN and IR Combination treatment). Cells containing >20 discrete foci per nucleus were scored as positive for damage or repair markers. ns = non-significant, *** *p* < 0.001, **** *p* < 0.0001.

The accumulation of DSBs indicates failed DNA repair and can compromise genomic integrity, leading to apoptosis [5,42]. Therefore, we assessed DSB formation and repair kinetics to determine whether the observed increase in apoptosis was due to persistent DNA damage. GBM cells were exposed to 5 Gy IR and assessed for γH2AX foci at time points corresponding to the initiation and completion of DSB repair (0, 1, 6, 24, and 48 h). An imbalance in dNTP levels is known to induce replication stress and cause spontaneous DNA damage [43]. Since elevating the dNTP pool could have a similar effect, we first measured γH2AX foci immediately after dN treatment and 48 h later. As shown in Supplementary Figure S4A,B, no significant increase in γH2AX foci was detected at 48 h post-dN treatment alone, indicating that elevating the level of all four dNTPs does not cause notable spontaneous DSBs and would not interfere with repair assessment. However, one hour after IR exposure, γH2AX foci were comparable with or without dN treatment, showing no interference with initial DSB induction (Figure 3A,B). Interestingly, by 24 h, γH2AX foci significantly decreased in cells treated with IR alone, whereas foci persisted up to 48 h in cells treated with the dN + IR combination (Figure 3C,D). These findings suggest that elevated dNTP levels delay DSB repair. To directly assess DSB repair efficiency, we performed a neutral comet assay following exposure to 5 Gy IR and a 24-h recovery period, during which the majority of DSBs are repaired. In this assay, cells with persistent DSBs have longer tail moments, indicating incomplete or delayed DNA repair [44]. Consistent with our γH2AX findings, cells treated with the dN + IR combination displayed significantly longer tail moments 24 h post-IR exposure, providing a direct demonstration that elevated dNTP levels impair DSB repair efficiency (Figure 3E,F and Supplementary Figure S4C).

### 2.4. Elevated dNTP Pool Levels Impair HR-Mediated DSB Repair by Promoting DNA Pol α Engagement at a Damage Site

**Figure 4:**
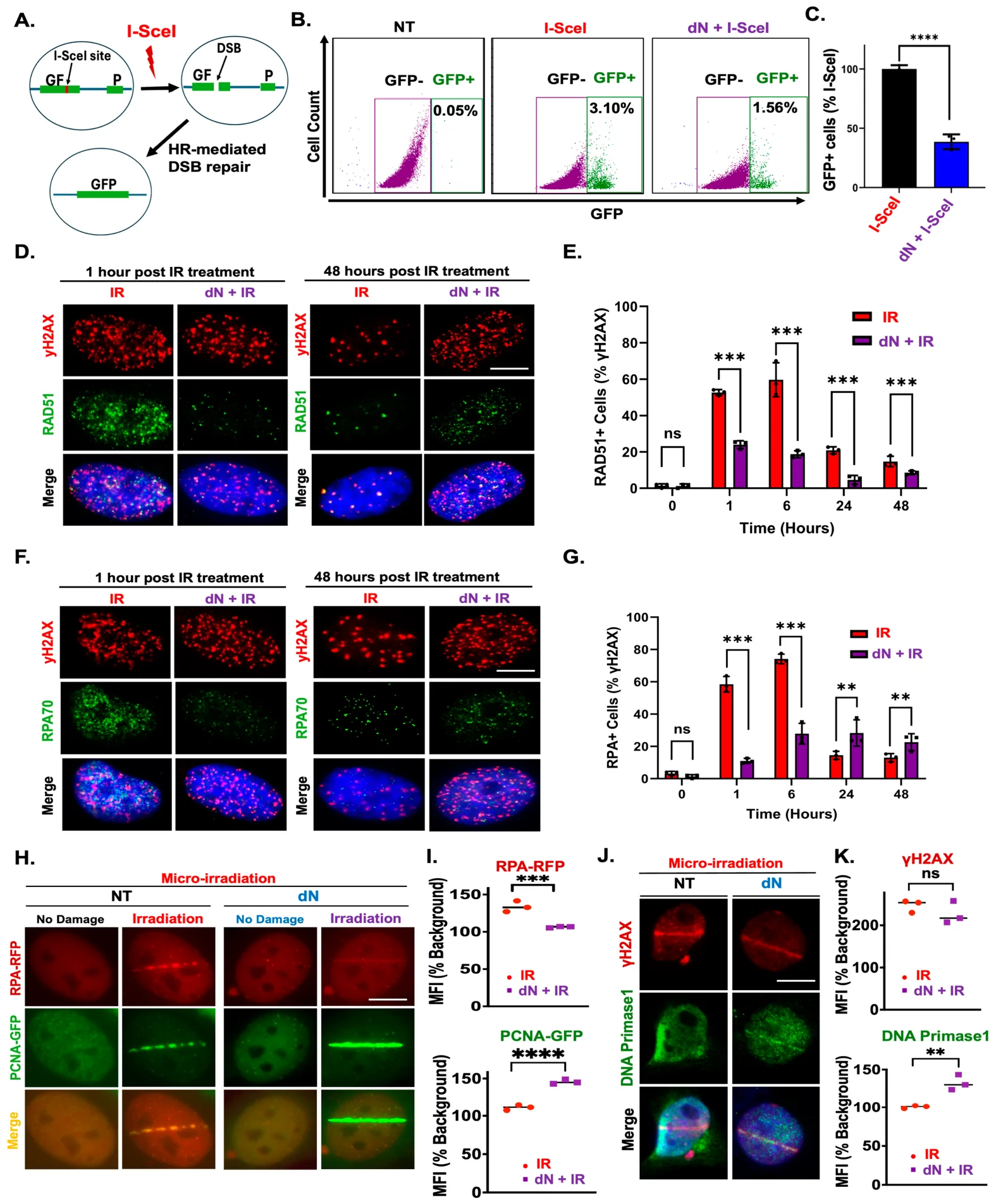
**Impaired DNA end resection reduces HR-mediated repair in GBM cells with elevated dNTP levels.** (**A**): Schematic of the Homologous Recombination GFP Reporter gene assay that induces DSB by I-SceI expression. (**B**,**C**): Cells with an integrated, disrupted GFP reporter gene were transfected with I-SceI, with or without dN treatment, harvested, fixed, and analyzed with flow cytometry. (**B**): Representative panels and (**C**): Quantification. Quantification is the percent I-SceI (Student’s *t*-test). (**D**): γH2AX (red), RAD51 (green), and DAPI (blue) immunofluorescence staining representative cell images at 1 h and 48 h post 5Gy IR treatment with or without dN treatment. Scale bar, 50 µm. (**E**): DAPI was used to quantify total cells, from which damaged cells (γH2AX-positive) were quantified. RAD51-positive cells were then quantified from γH2AX-positive cells at 0, 1, 6, 24, and 48 h post-5Gy IR with or without dN treatment (Student’s *t*-test). (**F**): γH2AX (red), RPA70 (green), and DAPI (blue) immunofluorescence staining representative cell images at 1 h and 48 h post-5Gy IR treatment with or without dN treatment. Scale bar, 10 µm. (**G**): DAPI was used to quantify total cells, from which damaged cells (γH2AX-positive) were quantified (Student’s *t*-test). RPA70-positive cells were then quantified from γH2AX-positive cells at 0, 1, 6, 24, and 48 h post-5Gy IR with or without dN treatment. (**H**): Laser microirradiation assay of live cells expressing PCNA-GFP and RPA-RFP fusion proteins at aligned DSB lesions with and without dN treatment. Scale bar, 10 µm. (**I**): Mean fluorescent intensity of the RPA-RFP and PCNA-GFP localization to the microirradiation line 1 h after damage. Data shown are from three nuclei (Student’s *t*-test). (**J**): Endogenous γH2AX (red), PRIM1 (green) localization at DSB lesion from laser micro-irradiation with or without elevated dNTP levels 1 h after damage. Scale bar, 10 µm. (**K**): Mean fluorescent intensity of γH2AX and PRIM1 localization to the microirradiation line 1 h after damage (Student’s *t*-test). In (**I**,**K**), 100% represents the background signal. Cells containing >20 discrete foci per nucleus were scored as positive for damage or repair markers. ns = non-significant, ** *p* < 0.01, *** *p* < 0.001, **** *p* < 0.0001.

Homologous recombination (HR) allows for the accurate repair of DSBs, and inhibiting HR can make GBM more vulnerable to IR and TMZ [45]. Since HR involves extensive DNA end processing, it could be affected by altered dNTP pool levels. Therefore, we determined whether the observed decrease in DSB repair under elevated dNTP levels, as shown in Figure 3, results in inefficient HR. To this end, we utilized an HR reporter gene assay in cells carrying a disrupted *GFP* gene containing an I-SceI site. After transfection with I-SceI to introduce site-specific DSBs, only successful HR-mediated repair restores disrupted GFP expression (schematics presented in Figure 4A). Comparable RPA70-GFP overexpression with or without dN treatment confirmed that elevated dNTP levels do not hinder transfection efficiency or protein expression, the two factors crucial for the reporter gene assay (Supplementary Figure S5A,B). Interestingly, as shown in Figure 4B,C and Supplementary Figure S5C,D, the percentage of GFP-positive cells was significantly lower following dN treatment and I-SceI transfection. This demonstrates a significant reduction in HR-mediated repair under elevated dNTP conditions. The titration shows that impaired HR is related to the concentration of dN (Supplementary Figure S5E,F). To determine which HR phase was affected, we monitored the recruitment of key proteins to DSB sites following IR exposure. Localization of RAD51, which is essential for strand invasion, to DSB sites was significantly reduced in cells treated with dNs before IR exposure (Figure 4D,E). Next, we investigated the recruitment of RPA70, a critical protein that binds to single-stranded DNA generated during end resection [14]. Consistent with impaired RAD51 recruitment, RPA70 localization was significantly reduced under elevated dNTP levels, suggesting a disruption in DNA end resection (Figure 4F,G). Additionally, the localization of 53BP1, a mediator of DSB signaling and repair choice [19], was significantly reduced (Supplementary Figure S5G,H). Because 53BP1 is a potent inhibitor of DNA end resection, a crucial step in HR progression, this finding, together with dNTP-triggered delayed G2/M progression shown in Figure 2B–E, indicates that the HR pathway is preferred under elevated dNTP levels [46]. However, the impaired RPA and RAD51 mobilization to the DSB site suggests that there is limited single-stranded DNA available for homology search, a crucial step for HR to proceed. This prompted us to examine the potential factor. Recent studies have provided evidence of DNA Polymerase α (DNA Pol α) engagement at resected DNA ends. Additionally, it has been demonstrated that DNA Pol α initiates synthesis on processed 3′ overhangs, thereby limiting the resection of DSB ends and their length, and counteracting exonuclease activity [19,46,47]. Therefore, we examined whether elevated dNTP levels promote aberrant DNA Pol activity at sites of DNA damage. To this end, we used laser microirradiation to determine the spatiotemporal dynamics of polymerase engagement at DSB lesions by measuring PCNA recruitment (as a proxy for polymerase engagement [48]). Under elevated dNTP conditions, significantly increased PCNA-eGFP accumulation was observed at the micro-irradiated sites and persisted for up to two hours, while under normal dNTP levels, it was weaker and lasted for a shorter period (30 min) (Figure 4H,I, Supplementary Figure S5I). Concurrently, RPA70-RFP recruitment was significantly diminished, consistent with previous results presented in Figure 4F,G. Impaired RPA localization, combined with increased PCNA accumulation, is indicative of augmented DNA Pol α activity, which could interfere with the generation of single-stranded DNA overhangs required for HR [19,21]. Since the orientation of resected DNA does not permit the canonical 5′ to 3′ synthesis, priming is required to initiate DNA Pol α activity. To overcome this limitation, DNA Pol α forms a holoenzyme with DNA primase 1 (PRIM1), mimicking lagging-strand DNA synthesis [49]. Therefore, we probed for recruitment of endogenous PRIM1 along with γH2AX. Interestingly, PRIM1 localization at the DNA damage sites was observed exclusively in cells with elevated dNTP levels (Figure 4J,K), suggesting aberrant engagement and potential gap-filling synthesis. These findings support a model in which elevated dNTP levels promote premature single-stranded DNA end synthesis during resection, disrupting proper exonuclease-mediated processing, consistent with impaired RPA70 and RAD51 accumulation at the DNA damage site and delayed DSB repair presented in Figure 3.

### 2.5. Pol α Inhibition Rescues Impaired HR Associated Proteins Recruitment to the DNA Damage Site and DSB Resolution Under Elevated dNTP Levels

**Figure 5:**
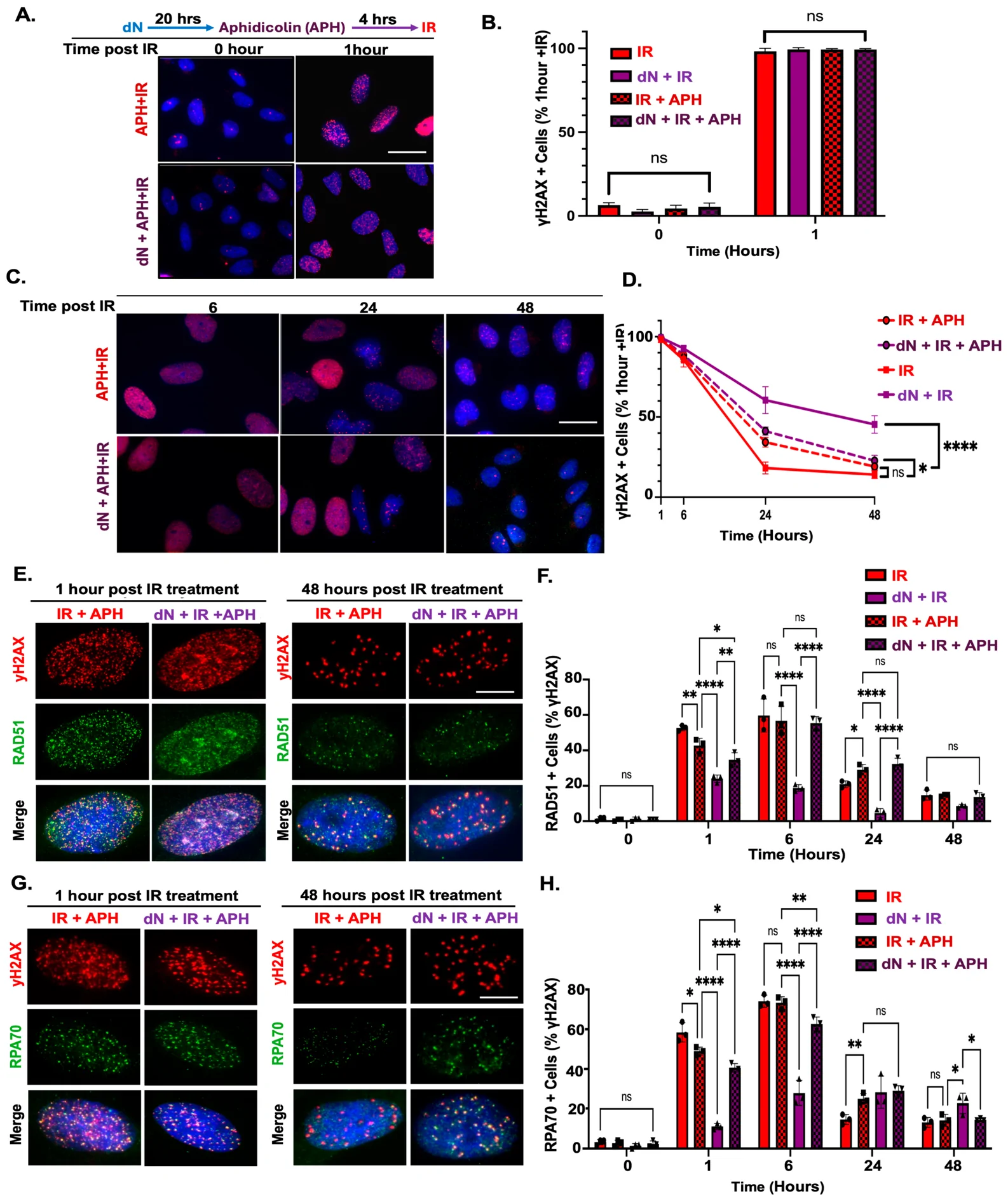
**Inhibiting *****DNA*** ***Pol α*** **restores RAD51 and RPA70 recruitment and resolves persistent DSBs under elevated dNTP conditions.** (**A**): γH2AX (red) foci 48 h after aphidicolin (APH) pretreatment (5 µM) (time 0) or APH + dN treatment in LN229 and 1 h post 5 Gy IR. Scale bar, 50 µm. (**B**): Quantification of 0-h and 1-h γH2AX foci with the indicated treatment condition (Two-Way ANOVA). (**C**): γH2AX (red) foci 48 h after aphidicolin (APH) pretreatment (5 µM) (time 0) or APH + dN treatment in LN229 and 6-, 24-, and 48-h post 5 Gy IR. Scale bar, 50 µm. (**D**): Quantification of 1-, 6-, 24-, and 48-h γH2AX foci with the indicated treatment (Two-Way ANOVA). (**E**): Representative immunofluorescence images showing γH2AX (red), RAD51 (green), and DAPI (blue) in LN229 cells treated with APH + IR vs. dN + APH + IR at 1 and 48 h. Scale bar, 10 µm. (**F**): Quantification of RAD51 and γH2AX foci across a 48-h time course under APH + IR and dN + APH + IR conditions (Two-Way ANOVA). (**G**): Representative immunofluorescence images of RPA70 (green), γH2AX (red), and DAPI (blue) in LN229 cells 1 and 48 h after IR ± dN under APH treatment. Scale bar, 10 µm. (**H**): Quantification of RPA70 and γH2AX foci over time for APH + IR and dN + APH + IR treatments (Two-Way ANOVA). Cells containing >20 discrete foci per nucleus were scored as positive for damage or repair markers. ns = non-significant, * *p* < 0.05, ** *p* < 0.01, **** *p* < 0.0001.

Our results demonstrate that elevated dNTP levels impair RAD51 and RPA70 recruitment to DSB sites and increase PCNA and DNA PRIM1 localization. These findings suggest abnormal engagement of the DNA Pol α complex with resected DNA ends, disrupting proper DNA end processing. To determine whether this effect depends on polymerase α activity, we treated cells with aphidicolin (APH), a well-defined inhibitor of DNA pol α [50]. APH treatment alone or dN and APH combination treatment did not induce notable DNA damage (negligible γH2AX foci) (Figure 5A,B). However, a similar level of γH2AX induction was observed one hour after IR treatment with APH alone (APH + IR) or dN and APH treatment (dN + APH + IR). In contrast, as previously shown (Figure 3C,D), dN-treated cells were unable to resolve the induced γH2AX 24 and 48 h later. APH rescued this phenotype, allowing the cells to resolve γH2AX at a rate similar to that observed with IR alone or with IR and APH (Figure 5E,F), indicating that Pol α activity contributes to the DSB repair defect observed under elevated dNTP conditions. Furthermore, in dN-treated cells, APH treatment restored both RAD51 and RPA70 localization to the DSB site (Figure 5G,H), strongly suggesting that end resection and single-stranded DNA generation were recovered. These findings support a model in which DNA Pol α activity impairs HR by limiting the extent of single-stranded DNA formation.

### 2.6. Elevated Cellular dNTP Levels Resensitize Patient-Derived Therapy-Resistant GBM Cells to TMZ and IR

**Figure 6:**
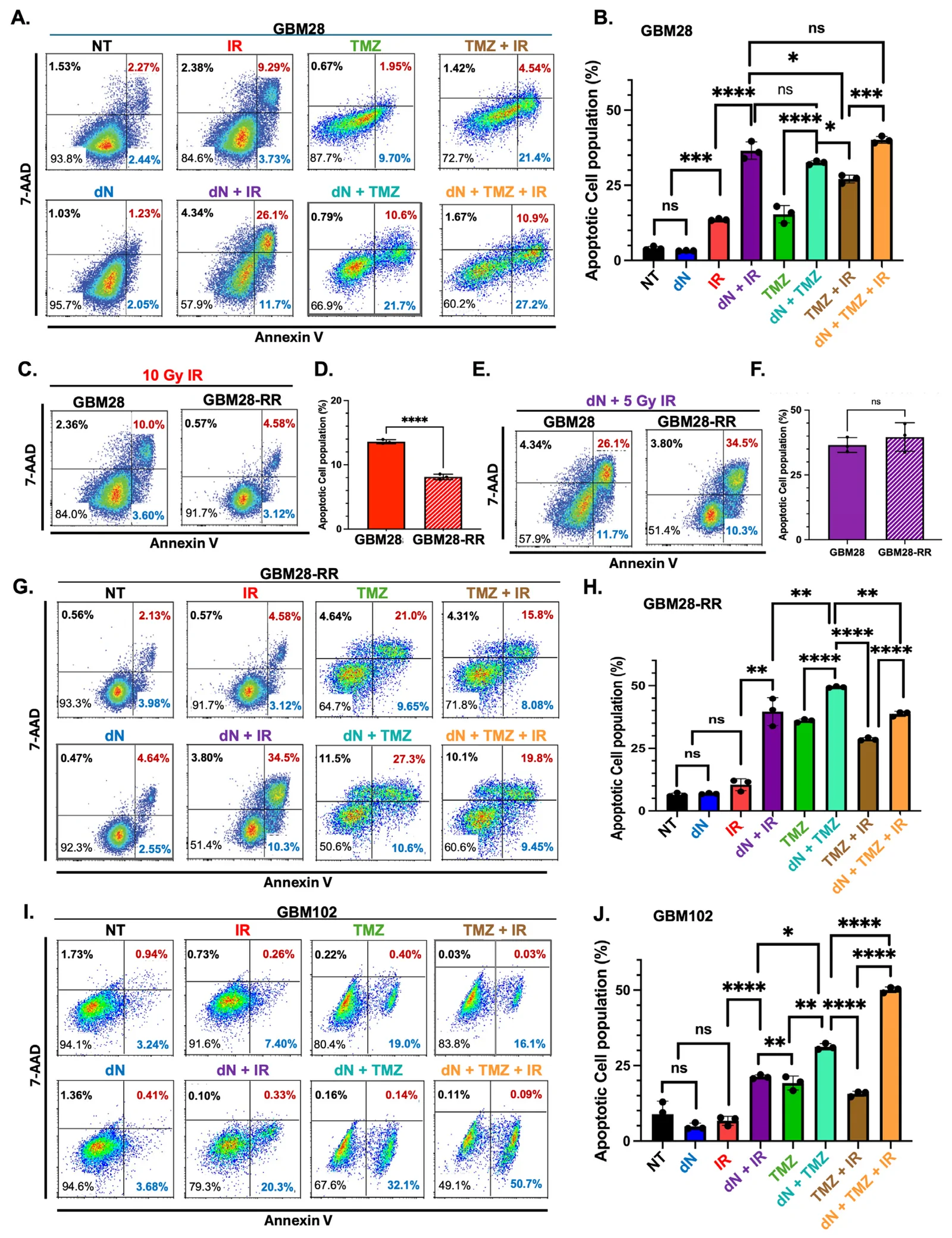
**dNTP elevation overcomes genotoxic therapy resistance in GBM.** Annexin V/7AAD staining of GBM cells to determine early (Quadrant 4-Blue) and late-stage apoptosis (Quadrant 2-Red) and necrosis (Quadrant 1-Black) (**A**): Annexin V/7AAD staining of GBM28 cells 48 h after the indicated treatment to determine early and late-stage apoptosis. Repeated N = 2, with a sample size of N = 3. (**B**): Quantification of total apoptotic GBM28 cell percentage (One-Way ANOVA). (**C**): Annexin V/7AAD staining of GBM28-RR primary cells compared with the parental cells 48 h after 10 Gy IR. (**D**): Quantification of total apoptotic cell percentage (Student’s *t*-test). (**E**): Annexin V/7AAD staining of GBM28-RR primary cells compared with the parental GBM28 primary cell line 48 h after 0.5 mM dN + 5 Gy IR. (**F**): Quantification of total apoptotic cell percentage (Student’s *t*-test). (**G**): Annexin V/7AAD staining of GBM28-RR primary cells 48 h after the indicated treatment. (**H**): Quantification of total apoptotic and necrotic cell percentage (One-Way ANOVA). (**I**): Annexin V/7AAD staining of recurrent GBM102 cells 48 h after the indicated treatment. (**J**): Quantification of total apoptotic cell percentage (One-Way ANOVA). ns = non-significant, * *p* < 0.05, ** *p* < 0.01, *** *p* < 0.001, **** *p* < 0.0001.

While established cell lines are useful for mechanistic studies, patient-derived models better represent the disease. Therefore, we tested whether elevated dNTP levels could sensitize GBM28, a patient-derived cell line obtained from the Mayo Clinic Brain Tumor Patient-Derived Xenograft National Resource. Consistent with our results from established cell lines (Figure 1D–G and Supplementary S1A,B), GBM28 showed significantly reduced cell growth and viability when treated with dN and IR (Supplementary Figure S6A). As observed in LN229 (Figure 2G,H), dN + IR and dN + TMZ combination treatments resulted in significantly greater apoptosis than IR or dN treatment alone (Figure S6A,B). These sensitizing effects were further validated in a second patient-derived line, GBM12 (Supplementary Figure S6C–F). Notably, GBM28 harbors an unmethylated *MGMT* promoter [27,28,29], rendering it highly resistant to TMZ-induced apoptosis under normal dNTP conditions (Figure 6A,B). However, it showed significantly increased sensitivity following dN + TMZ combined treatment, demonstrating the relevance of elevated dNTP levels beyond IR. Interestingly, the combination of dN + TMZ + IR induced similar levels of apoptosis as dN + TMZ or dN + IR (Figure 6A,B). These results emphasize the relevance of dNTP pool levels to the efficacy of standard-of-care treatment in GBM. Following multiple rounds of genotoxic therapy, resistant cell populations emerge, promoting recurrence, and this remains a significant challenge. To address this, we generated an IR-resistant GBM28 subline (GBM28-RR) through fractionated IR exposure as described in Supplementary Figure S6H and tested whether the dN-mediated increase in sensitivity to IR and TMZ can be recapitulated in these cells.

As shown in Supplementary Figure S6I,J, dN treatment alone did not induce significant apoptosis in GBM28-RR compared with the untreated group. However, following exposure to IR, it showed significantly decreased apoptosis compared with its parental line GBM28, confirming the successful induction of radio-resistance (Figure 6C,D). This is consistent with the known ability of GBM cells to develop resistance to therapy through adaptive mechanisms, including enhanced DNA damage response and shifts in metabolic pathways [29]. Interestingly, elevating the dNTP levels before IR treatment increased apoptosis in GBM28-RR to levels comparable to those in parental GBM28 cell lines under similar conditions (Figure 6E,F), demonstrating that elevating the cellular dNTP levels potentiates the sensitivity of highly resistant GBM cells to levels comparable to those of relatively less resistant cells. Despite unmethylated *MGMT*, GBM28-RR showed higher sensitivity to TMZ, which was further increased when combined with dN (Figure 6G,H, Supplementary Figure S6K). To test the relevance of the elevated dNTP levels to naturally occurring resistant cells, we tested GBM102, a cell line derived from a recurrent GBM tumor. As observed in GBM28-RR, there was no significant difference in apoptosis when GBM102 was treated with IR alone, showing its resistance, but it showed some sensitivity to TMZ (Figure 6I,J and Supplementary Figure S6G). Combination treatment of dN with IR or TMZ significantly increased apoptosis, demonstrating the importance of elevated dNTP levels for naturally occurring resistant GBM cells (Figure 6I,J). Interestingly, unlike GBM28-RR, the naturally occurring GBM102 showed a three-fold increase in sensitivity when dN was combined with both IR and TMZ (dN + IR + TMZ) compared with the standard-of-care treatment IR + TMZ combination (Figure 6I,J). Findings from this study demonstrate that elevating dNTP levels before IR, TMZ, or IR + TMZ can disrupt survival pathways, overcoming genotoxic resistance and significantly increasing sensitivity, even in highly resistant and recurrent GBM. These observations support our finding that elevated dNTP impairs DNA repair by promoting the synthesis of resected DNA required for HR.

## 3. Discussion

A significant obstacle to improving cancer treatment outcomes is overcoming therapy resistance. Furthermore, repeated exposure to treatments can unintentionally apply selective pressure, leading to the emergence of more resistant cancer cells [33,51]. Dysregulated DNA repair pathways, particularly HR, are a hallmark of genotoxic therapy-resistant cancers. While targeting DSB repair-associated proteins has shown promising results, there has been no significant progress in improving the survival of GBM patients over the last two decades [6,7]. Our findings reveal a novel mechanism by which elevating the intracellular dNTP levels disrupts homologous recombination and impairs DNA repair following exposure to IR, TMZ, or both. This disruption drives apoptosis even in highly resistant GBM cells.

Our findings align with previous studies, demonstrating that dNTP imbalances can interfere with genomic stability and DNA repair fidelity [22]. However, conventional approaches have primarily focused on depleting the cellular dNTP levels or modulating a single nucleotide. In contrast, this study uniquely highlights the consequences of elevating all four cellular dNTP levels, thereby filling a crucial gap in our understanding of GBM resistance mechanisms. Furthermore, we increased all four dNTP levels proportionally, thereby avoiding unintended effects that could lead to mutations [22], as confirmed by RNA sequencing data presented in Supplementary Figure S2A. However, further study is required to fully understand the consequences of elevated dNTP levels, particularly following IR exposure. IR could oxidize bases, particularly guanine, generating 8-oxodG, which could impact mutation and DNA synthesis. This investigation could be extended to the repair of such byproducts by corrective enzymes like OGG1 [52]. This study provides new insights into overcoming therapy resistance in GBM.

Our study also builds on a recent discovery of a key mechanism that implicates Pol α in HR-mediated DSB repair. This study demonstrated that Pol α can engage and initiate the synthesis of end-resected DNA following induced DSBs [19,21], an interesting discovery that introduces a new dynamic in DNA repair, particularly HR. Our data suggest that when the dNTP levels are increased before DSB induction, Pol α is recruited to the DSB site. Additionally, the recruitment of PCNA further supports the initiation of DNA synthesis at the site of damage. The observed recruitment of DNA pol α was accompanied by the impaired recruitment of RPA70 and RAD51 at DSB sites, suggesting that DNA pol α-mediated gap-filling synthesis directly undermines homologous recombination by limiting the extent of end resection required for efficient HR [13,53]. This functional linkage was strongly supported by our rescue experiment, in which inhibition of DNA pol α restored RPA70 and RAD51 recruitment and accelerated DSB resolution under elevated dNTP conditions. Our results support a model in which elevated dNTP levels impair HR via aberrant polymerase activity during resection. This impairment of HR resulted in the accumulation of DSBs, cell-cycle arrest, and activation of apoptotic pathways leading to cell death. While our findings provide strong evidence that elevated dNTP levels impair HR by promoting Pol α-dependent interference with DNA end resection, our conclusions remain indirect. Future studies using SAR-seq or END-seq, as employed by Paiano et al. to demonstrate the synthesis of end-resected DNA [19], will be critical for quantifying gap-filling activity and directly validating the proposed mechanism. While investigating HR is crucial, DSBs can also be repaired through NHEJ, another major pathway that was not adequately addressed in this study. The decrease of 53BP1 accumulation at the damage site, as shown in Supplementary Figure S5G,H, suggests that elevated dNTP levels create an HR-favoring environment. This is interesting because, in an ideal situation, NHEJ dominates when HR is impaired. Our model shows that elevated dNTP levels favor HR-mediated repair, which is obstructed due to faulty end resection. Therefore, a systematic assessment of the NHEJ pathway is important for providing a full picture of the impact of dNTP pool levels on DSB repair.

The translational implications of our findings are significant. Current GBM tumor treatments rely heavily on radiation and chemotherapy; however, resistance remains a major challenge [9,10]. Our study suggests that modulating dNTP levels could be a novel strategy to sensitize GBM to the currently available standard-of-care treatments, even in the most resistant models. GBM cells derived from primary tumors (GBM28 and 12), which closely mimic the initial therapeutic response, showed significantly increased sensitivity to IR, TMZ, or IR/TMZ treatment when the dNTP levels were elevated. Furthermore, elevating dNTP levels substantially increased apoptosis in engineered IR-resistant GBM28 (GBM28-RR) and GBM102, a recurrent GBM model typically resistant to genotoxic treatments, reinforcing the robustness of this approach. Our findings suggest that elevated dNTP levels can overcome IR- and TMZ resistance in both engineered GBM28-RR and naturally recurrent GBM102. However, further research is necessary to fully understand the mechanisms behind resistance development in these cells. Importantly, our results emphasize the clinical relevance of MGMT promoter methylation. GBM28 cells possess an unmethylated MGMT promoter, which confers resistance to TMZ by enabling O6-methylguanine DNA methyltransferase activity to reverse the TMZ-induced DNA damage. Consistent with this, TMZ alone failed to induce significant apoptosis in GBM28. However, when dNTP levels were elevated, a significant apoptotic response was observed. These findings highlight the therapeutic potential of dNTP modulation to overcome intrinsic TMZ resistance, particularly in MGMT-unmethylated tumors, which are historically associated with poor outcomes. Future studies should investigate the interplay between dNTP modulation and other clinically relevant factors, such as *IDH* mutation status, patient age, and therapeutic history, to better define subsets of responsive patients. Additionally, although this is outside the scope of this study, the benefit of elevated dNTP levels in combination with other treatments that are not part of the GBM standard-of-care regimen should be evaluated. In light of these findings, the development of therapeutic approaches to modulate nucleotide metabolism appears promising. Further study is needed to confirm the approach’s practical application in clinical settings. Specifically, the treatment intervals and durations used to elevate cellular dNTP levels require more examination. This is crucial because these strategies are intended to be combined with existing treatments like IR and TMZ. Therefore, gaining a clear understanding of how cellular dNTP levels change over time is essential. Additionally, the specificity of the treatments should be evaluated by examining their effects on normal glial and neuronal cells and normal brain tissue.

Building on these findings, developing methods to therapeutically modulate dNTP levels is of great interest. While dN treatment is a potential candidate, one strategy to achieve elevated dNTP levels involves using drugs or gene therapy to target specific enzymes that control nucleotide metabolism. For example, we previously used a virus-like particle containing the viral protein-x (Vpx), which degrades SAMHD1, a potent dNTPase, in vitro [54,55] and thereby increasing the cellular dNTP levels. While it was effective in non-dividing cells like macrophages, SAMHD1 degradation alone did not significantly increase dNTP levels in actively dividing cells, such as T cells and cancer cells, likely because these cells already maintain high dNTP levels to support continuous DNA replication. Other targets, such as KAT7, should also be explored. KAT7 acetylates RRM2, disrupting its homodimerization. This will impair Ribonucleotide Reductase (RNR) function, thereby reducing dNTP synthesis [56]. Inhibiting or depleting KAT7 can enhance RNR activity, thereby increasing dNTP levels. Therefore, combining enhanced dNTP synthesis with SAMHD1 degradation may synergistically elevate dNTP levels to therapeutically relevant levels, offering a potential strategy for targeting DNA repair in cancer cells. Although not addressed in this study, preclinical animal models are crucial for assessing the practicality of the strategy developed based on the findings presented here for GBM treatment, particularly regarding delivery approach, selectivity, and ability to cross the blood–brain barrier.

## 4. Materials and Methods

### 4.1. Plasmids, Antibodies, and Reagents

The antibodies, plasmids, and other reagents used in this study are listed in Supplementary Tables S1–S3.

### 4.2. Cell Culture and Transfection

Glioblastoma cell lines LN229 (CRL-229) and U87 (BTB-14), and osteosarcoma cell line U2OS (HTB-96) were obtained from ATCC and grown in DMEM (Gibco, Waltham, MA, USA) supplemented with 10% FBS. GBM12 and GBM28 primary, and GBM102 recurrent patient-derived cells were obtained from the Mayo Clinic Brain Tumor Patient-Driven Xenograft National Resource and grown in DMEM (Gibco) supplemented with 2% FBS. All cells were incubated at 37 °C and 5% CO^2^, and the medium was changed every 3 days or as needed. Transfection was performed according to the manufacturer’s instructions using Lipofectamine 3000 (Thermo Fisher Scientific, Waltham, MA, USA) with a slight modification: a 1:1 ratio of Lipofectamine 3000 to p3000 reagent. Transfected cells were incubated at 37 °C in serum-free DMEM for 4 h and later replaced with FBS-supplemented DMEM. Cells were irradiated with a dose of 5 Gy, unless otherwise noted (X-RAD320 irradiator, Precision X-ray, Madison, CT, USA).

### 4.3. Apoptosis Assay and Cell Cycle Analysis

Annexin V-FITC and 7AAD (BioLegend, San Diego, CA, USA) were used according to the manufacturer’s instructions to measure apoptosis. Briefly, cells were harvested 48 h post-treatment, washed twice with ice-cold FACS buffer (PBS containing 2% FBS), resuspended in Annexin V binding Buffer, and stained with Annexin V and 7AAD. Flow cytometry was performed using NovoCyte’s Quanteon Flow Cytometer System and analyzed using FCS Express 7. Debris and doublets were excluded with strategic gating, and singlets were analyzed. Annexin V/7AAD staining was used to distinguish apoptotic/necrotic cells from live, healthy cells. For cell cycle analysis, the cells were washed twice in ice-cold PBS, fixed with ice-cold ethanol, permeabilized, and stained with propidium iodide (PI). The same gating criteria were used to eliminate cell debris and doublets. The MultiCycle AV module in FCS Express was used to determine cell cycle phase. The apoptosis assay was completed in biological triplicate twice. Except for 6C, 6E, and 6G, these were performed three times. The cell cycle analysis was conducted four times with biological triplicates.

### 4.4. Comet Assay

Cells were treated with dNs and irradiated as described above, trypsinized, washed with PBS, and embedded in low-melting agarose. The low-melting agarose containing the cells was cast onto a 1% agarose-coated slide, a coverslip was placed on it, and the slide was incubated at 4 °C for 30 min with N-tert-butyl-alpha-phenylnitrone (PBN-Sigma B7263, Sigma-Aldrich, St. Louis, MO, USA). After 30 min, the coverslips were removed, and the slide with the agarose gel was incubated in a buffer (0.5 M NaCl, 10 mM EDTA, 10 mM Tris, pH 10). Next, a 10.5% Triton X-100, 1×PBN, and 10% DMSO solution was added overnight at 4 °C. The following day, the slides were washed by submerging them in electrophoresis buffer for 5 min. Electrophoresis was performed in buffer (0.4 M Tris at pH 7.5) at 30 V (~0.74 V/cm) and 300 mA for 45 min. The slides were removed from the chamber, briefly submerged in distilled water, mounted with a DAPI coverslip, and left overnight. The slides were imaged the following day. The comet assay was conducted twice in biological triplicate; each sample had N = 50 nuclei scored with ImageJ (version 1.54).

### 4.5. dN Treatment and Quantification of Cellular dNTP Levels

The cellular dNTP level was increased by supplying deoxyribonucleosides (dNs), the unphosphorylated form of dNTPs. The dNs used include 2′-Deoxycytidine hydrochloride (Sigma-Aldrich, St. Louis, MO, USA, Sigma-D0776), 2′-Deoxyadenosine monohydrate (Sigma-D8668), Thymidine (Sigma-T1895), and 2′-Deoxyguanosine monohydrate (Sigma-D0901). The dNs were freshly prepared in PBS and added directly to the tissue culture media to reach the indicated concentration (Figure 1A). Cellular dNTP levels were measured using a previously described HIV-1 Reverse Transcriptase-based primer extension assay [30]. Briefly, the cells were treated with PBS or dNs. After 24 h, cells were washed with PBS, lysed in 65% aqueous methanol, and then heated for three minutes at 95 °C. Lysed samples were centrifuged at 15,000× *g* for 10 min; the pellet was discarded, and the supernatant was dried in a speed vacuum and then resuspended in nuclease-free water. The purified dNTP was used in the reaction to extend a single nucleotide at the 5′ end of the γ−^32^P-labeled 18-mer primer annealed to a 19-mer template with varying overhangs (T or G). The products were resolved on urea-PAGE, and the amounts of extended/non-extended primers were quantified using Bio-Rad Image Lab 6.1. The dNTP assay was performed twice, each in biological duplicate.

### 4.6. Western Blot

Cells were harvested and lysed on ice for 30 min in BC200 buffer (200 mM NaCl, 25 nM Tris-Cl (pH 8), 0.2% NP-40, 10% glycerol, 1 mM DTT, and 1 mM EDTA (pH 8)). The buffer was supplemented with protease inhibitors before each use. The protein concentrations were determined, resolved on an SDS gel, transferred to a PVDF membrane, and visualized using a LI-COR Odyssey infrared imaging system after probing with primary antibodies, followed by Alexa Fluor secondary antibodies (Life Technologies, Carlsbad, CA, USA). The Western blot analysis for DNA2, PARP1, and EXO1 was conducted in two separate experiments, with biological duplicates for each time point.

### 4.7. Generation of Cells with a Homologous Recombination Reporter Gene

The HR-reporter constructs were generated as previously described [57]. Briefly, the plasmid was linearized, resolved on an agarose gel, excised, and extracted using an Invitrogen PureLink Quick Gel Extraction Kit (Thermo Fisher Scientific (K210012)). Cells were grown to 70% confluency on a 10 cm plate and transfected with 0.5 μg of linearized plasmid using the aforementioned transfection protocol. Cells with an integrated reporter gene were selected with 1 mg/mL geneticin (G418) 24 h after transfection for 7 days. Successful integration was confirmed by visually assessing GFP expression under a microscope following transfection with a plasmid containing I-SceI. 96 h after I-SceI transfection, cells were harvested, fixed in 1% paraformaldehyde (PFA), and analyzed by FACS. Samples without I-SceI (No I-SceI) served as the GFP-negative control to set the gating for the GFP-negative cell population. Samples with I-SceI but no dN (I-SceI) served as the GFP-positive control to set the gating for the GFP-positive population. Signals above the negative control were considered GFP-positive. The GFP reporter gene assay was performed in biological triplicate, three times, except for the titration in Supplementary Figure S5, which was performed twice.

### 4.8. Generation of an Irradiation-Resistant Cell Line

Radiation-resistant cells (GBM28-RR) were generated according to a previously described protocol [58]. Briefly, GBM28 primary cells were exposed to a fractionated radiation regimen weekly (X-RAD320 irradiator, Precision X-ray, Madison, CT, USA). An initial dose of 2 Gy was followed by incremental weekly doses of 0.5 Gy for 12 weeks and a total radiation dose of 60 Gy. The cells were subsequently maintained with further weekly doses of 5 Gy (Supplementary Figure S5H). The resistance was confirmed by exposing the cells to 10 Gy IR.

### 4.9. Cell Viability and Clonogenicity Assay

For the cell viability assay, the cells were seeded in triplicate at a density of 10^3^ cells/well in a 96-well plate. After 4 h, the medium was removed, and fresh medium containing 0.5 mM dNs was added, as indicated. After 24 h, the medium was changed, and the cells were irradiated. Cells were allowed to recover for 48 h, and viability was assessed using 10% AlamarBlue (Thermo Fisher DAL1100). The fluorescence signal was measured at 540 nm excitation and 590 nm emission, and the viability fractions were normalized to untreated controls exposed to identical transfection conditions. For the clonogenicity assay, cells were seeded at a density of 300 cells/well in triplicate and treated with dNs. dN-treated and non-treated cells were exposed to IR as described above and grown for two weeks, or until the majority of colonies reached 50 cells or above. The colonies were fixed, stained with crystal violet, and scored. The surviving fraction was determined by the following formula: The number of colonies formed/(The number of cells seeded x The plating efficiency). Individual colonies were scored using UVP ChemStudio, Analytikjena (Jena, Germany). The Alamar Blue and clonogenic assays were completed 3 times, in biological triplicate each time.

### 4.10. Immunocytochemistry (Immunofluorescence Assay)

Cells were seeded on coverslips, incubated with dNs as described above, and irradiated. After 1-, 6-, 24-, and 48-h recovery, cells were washed with PBS, fixed with 4% PFA, and incubated with 0.5% Triton X-100 at room temperature for permeabilization. The cells were blocked with 5% BSA in PBS, incubated with primary antibodies at 4 °C overnight, and probed with Alexa Fluor 488 (Molecular Probes, Eugene, OR, USA) (green) or 555 (red) secondary antibodies. The coverslips were mounted with DAPI Fluoromount-G (Southern Biotech, Birmingham, AL, USA) and analyzed with a Zeiss Axio Observer 7 microscope (Carl Zeiss Microscopy, Jena, Germany) equipped with Axiocam and controlled by Zeiss Zen 2.6 software. Images were obtained with 40× oil-immersion objectives. For each condition and time point, 50 cells were counted in triplicate based on DAPI staining, and >20 γH2AX foci were used as an indicator of damage. To rescue the impaired γH2AX, RPA70, or RAD51 recruitment, cells were treated with 5 μM APH (Sigma-A0781, St. Louis, MO, USA) for 4 h, with or without dN treatment, and exposed to 5 Gy IR. IF experiments were conducted in biological triplicate three times, where 50 foci were counted in 3 randomly selected sections of each slide for each condition.

### 4.11. Laser Microirradiation

Cells were plated on a glass-bottom dish (Southern Labware, Cumming, GA, USA) and transfected with PCNA-eGFP and RPA-RFP. Transfected cells were treated with dN as indicated above, and protein mobilization to the damage site was conducted on live cells. Time-lapse images were taken to capture the recruitment of the protein of interest using a Zeiss Axio Observer 7 microscope (Carl Zeiss Microscopy) controlled by Zeiss Zen 2.6 software and equipped with a heated chamber and UGA-42 Geo Caliburn Micro-irradiator (Rapp Optoelectronics-89-North, Wedel, Germany): 355 nm; 1-kHz repetition rate; 3-ns pulse, and the laser power set at 50%. PCNA-GFP mobilization was monitored, and RPA-RFP was used as a positive control for successful DSB induction. Endogenous PRIM1 (the catalytic subunit of the DNA primase complex and component of the Pol α complex, respectively) was monitored by immunocytochemistry, and endogenous γH2AX was used as a positive control for successful DSB induction. To assess mobilization of endogenous proteins, the cells were fixed in 4% paraformaldehyde following microirradiation, permeabilized with 0.5% Triton X-100, and blocked with 5% BSA. Primary antibodies against γH2AX and PRIM1 were added to the glass-bottom dish and incubated overnight at 4 °C. Then, the plates were washed with PBS and incubated with secondary antibodies conjugated to Alexa Fluor dyes. Nuclei were stained with DAPI. Images were acquired using the Zeiss Axio Observer 7 microscope and analyzed using the Zeiss Gen 2.6 software. Exogenous laser experiments were done on multiple cells in the same well. This was done 4 times per condition. Endogenous laser experiments were done on multiple cells in the same well. Repeated in duplicate per condition.

### 4.12. RNA Sequencing

Total RNA was purified from cells using the PureLink RNA Mini Kit per the manufacturer’s instructions (Invitrogen, Waltham, MA, USA) 48 h after the indicated treatments. RNA sequencing was performed by MedGenome (Foster City, CA, USA). The reference genome (hg38) and gene model annotation files from Ensembl were used to align the reads using RNA-STAR. The reads were quantified using FeatureCounts in unstranded mode. The DESeq2 package in RStudio (version 4.4.1) was used to identify differentially expressed genes, and an absolute fold change (FC) ≥ 1.5 with an adjusted *p*-value (*p*. adj) < 0.05 was considered significant. For mutation analysis, STAR-aligned BAM files were processed in the Galaxy platform (Usegalaxy.org) using FreeBayes to call variants and generate VCF files for each sample. The VCF files were then imported into R (version 4.6.1) using the VariantAnnotation package, where genotype calls were extracted, and non-reference allele counts were determined by excluding the homozygous reference (“0/0”) and missing (“./.”) calls. These data were subsequently used to quantify the mutation burden across treatment groups and were visualized using ggplot2. The RNA-seq was done once in biological triplicate.

### 4.13. Statistical Analysis

Statistical analyses were conducted using GraphPad Prism 10 software. All Student’s *t*-tests were unpaired, and *p*-values ≤ 0.05 (with 95% confidence intervals) were considered statistically significant. In cases involving multiple group comparisons, one-way or two-way ANOVA was followed by appropriate post hoc tests to assess statistical significance. Continuous outcome data are presented as the mean ± standard error of the mean (SEM), and error bars represent the SEM. For all box-and-whisker plots, the whiskers represent the minimum and maximum values, the lower and upper box edges correspond to the 25th and 75th percentiles, respectively, and the lines within the boxes indicate the median. Significance is noted as: ns = non-significant, * *p* < 0.05, *p* < 0.01, *** *p* < 0.001, **** *p* < 0.0001.

## 5. Conclusions

This study demonstrates that elevating cellular dNTP pool levels promotes DNA Pol α engagement at DSBs and impairs DNA end-resection. Our findings further reveal that HR-mediated repair is significantly reduced, exposing a promising vulnerability in therapy-resistant GBM and other solid cancers. These findings underscore the importance of further research into the interplay between intracellular dNTP levels and DSB repair, as well as its potential applications in cancer treatment.

## Data Availability

The RNA-seq data presented in the study are openly available in [GSE346705]. The original contributions presented in this study are included in the article. Further inquiries can be directed to the corresponding author.

## Supplementary Materials

The following supporting information can be downloaded at: https://www.mdpi.com/article/10.3390/ijms27188045/s1.
