# Supplementary material for: Elevated dNTP Pool Levels Impair Homologous Recombination and Enhance Glioblastoma Sensitivity to Irradiation and Temozolomide
Source: Int J Mol Sci. 2026 Sep 10;27(18):8045. doi: 10.3390/ijms27188045 (PMC13606881; doi:10.3390/ijms27188045)
Supplement: Supplementary file 1 [file ijms-27-08045-s001.zip › ijms-4529973-supplementary.pdf]

International Journal of *Molecular Sciences*

MDPI

Supplemental Material

Elevated dNTP Pool Levels Impair Homologous Recombination and Enhance Glioblastoma Sensitivity to Irradiation and Temozolomide

Dominique Monroe [1,2], Mercy Kehinde-Ige [1,2,3], Arilyn Williams [1,2], Vafa Ismayilova [1,2], Apeksha Anand [2,3], Matthew Kededa [1,2], Ramsha Khanam [1,2], Aman Kalsi [1,2], Ali S. Arbab [1,2], Daitoku Sakamuro [1,2], Huidong Shi [1,2,3] and Waaqo Daddacha [1,2,*]

[1] Department of Biochemistry and Molecular Biology, Medical College of Georgia, Augusta University, Augusta, GA 30912, USA; dominique.monroe@vai.org (D.M.); mkehindeige@augusta.edu (M.K.-I.); awilliams33@augusta.edu (A.W.); vismayilova@augusta.edu (V.I.); mkededa@augusta.edu (M.K.); rkhanam@augusta.edu (R.K.); akalsi@augusta.edu (A.K.); aarbab@augusta.edu (A.S.A.); dsakamuro@augusta.edu (D.S.); hshi@augusta.edu (H.S.)

[2] Georgia Cancer Center, Augusta University, Augusta, GA 30912, USA; apanand@augusta.edu

[3] Immunology Center of Georgia, Augusta University, Augusta, GA 30912, USA

* Correspondence: wdaddacha@augusta.edu

Table S1. List of Antibodies.

Name	Species	Company	Catalog #
GAPDH	Mouse	Santa Cruz	sc-47724
GAPDH	Rabbit	Cell Signaling	2118S
RPA70	Rabbit	Cell Signaling	2267S
γH2AX	Mouse	Millipore Sigma	05-636
RAD51	Rabbit	Millipore Sigma	PC130100L
PRIM-1	Rabbit	Proteintech	10773-1-AP
EXO1	Rabbit	Invitrogen	PA5-12501
DNA2	Rabbit	Proteintech	18727-1-AP
53BP1	Rabbit	Bethyl	A300-273A
PARP	Rabbit	Invitrogen	PA5-16356

Table S2. List of Plasmids.

Name	Company	Catalog #
pcDNA3.1(+) eGFP	Addgene	129020
pcDNA3.1-RFP	Addgene	206177
PCNA-eGFP	Addgene	104243
RPA-RFP	Engineered in lab	N/A
I-SceI expression plasmid	Addgene	26477

https://doi.org/10.3390/ijms27188045

Table S3. List of Reagents.

Name	Company	Catalog #
2'-Deoxyadenosine monohydrate	Millipore Sigma	D8668
2'-Deoxythymidine monohydrate	Millipore Sigma	T1895
2'-Deoxyguanosine monohydrate	Millipore Sigma	D0901
2'-Deoxycytidine hydrochloride	Millipore Sigma	D0776
ATP, [γ-32P]-6000Ci/mmol, 250 μCi	Revvity	BLU0027250UC
Temozolomide	Millipore Sigma	T2577-25MG
Alamar Blue	Thermo Fisher Scientific	DAL1100
Annexin V / 7AAD	Bio-Legend	640922
Aphidicolin (APH)	Sigma	A0781
Lipofectamine 3000	Thermo Fisher Scientific	L3000015
Propidium Iodide (PI)	Thermo Fisher Scientific	P3566
DAPI Fluoromount-G®	SouthernBiotech	0100-20
PureLink RNA Mini Kit	Invitrogen / Thermo Fisher Scientific	12183018A
Geneticin (G418)	Gibco / Thermo Fisher Scientific	10131035

https://doi.org/10.3390/ijms27188045

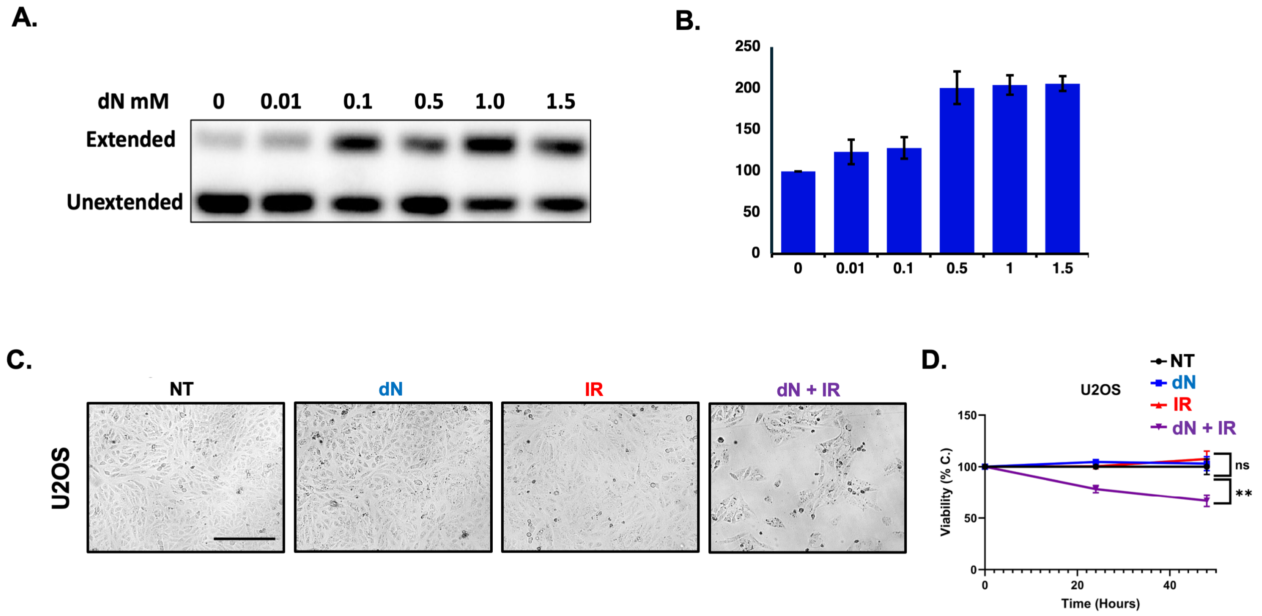

Supplemental Figure S1: **Cell viability of osteosarcoma cell line with elevated dNTP level. A-B:** HIV-1 RT based dNTP assay determining the correlation between the concentration of supplemented dNTP and cellular dNTP pool level increment. (A) Representative image and (B) quantification. **C**: Bright-field representative images of U2OS cells 48 hours after the indicated treatment. **B:** Cell viability quantified by Alamar Blue staining 24 and 48 hours after treatment (Two-Way ANOVA). Scale bar: 400 μm

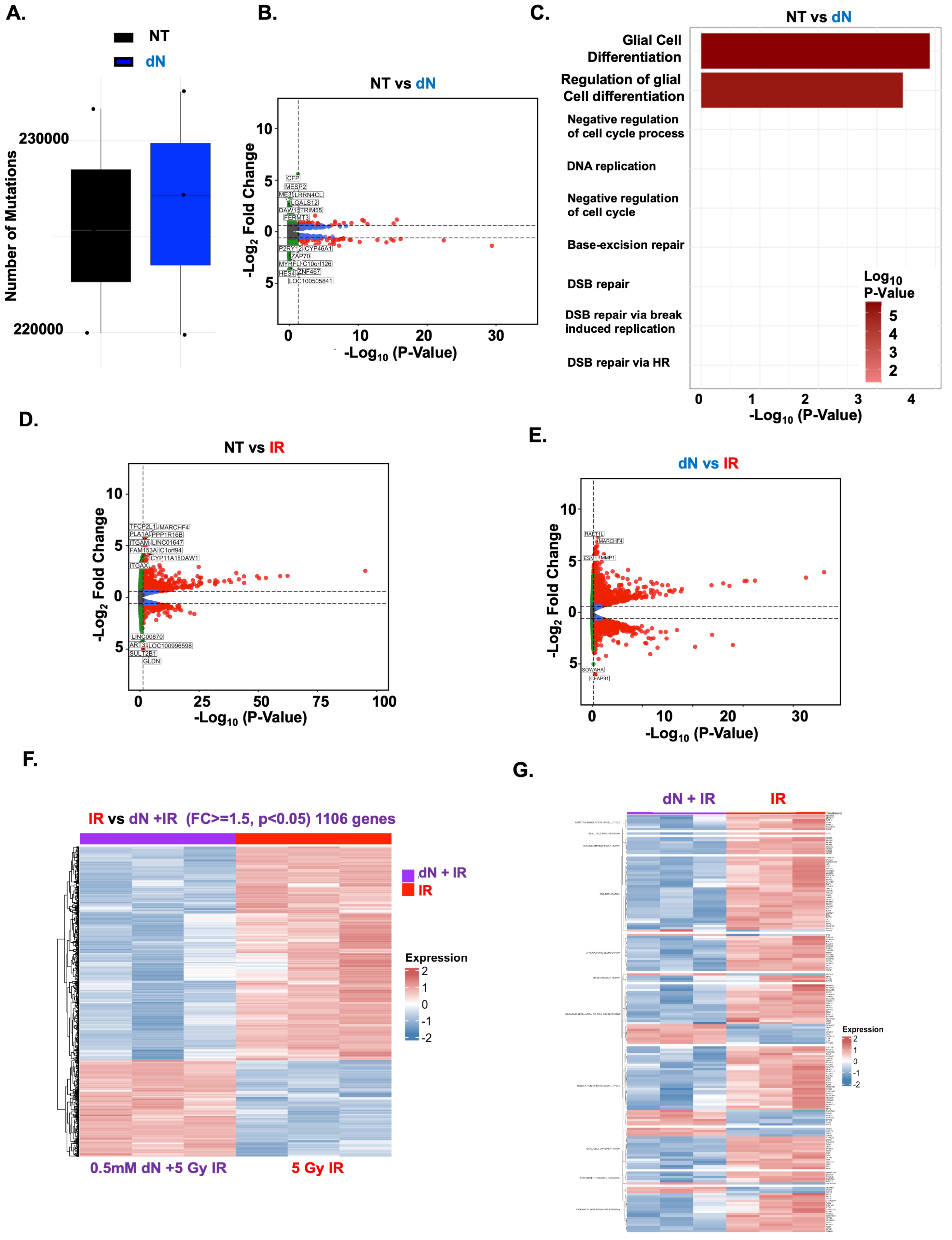
A.
NT
dN
Number of Mutations
230000
220000
B.
NT vs dN
-Log2 Fold Change
-Log10 (P-Value)
CFP
MESP2
LRRN4CL
LGALS12
DAW1
TRIM55
FERMT3
P2RY12
CYP46A1
ZAP70
MYRFL
C10orf126
HES4
ZNF467
LOC100505841
C.
NT vs dN
Glial Cell Differentiation
Regulation of glial Cell differentiation
Negative regulation of cell cycle process
DNA replication
Negative regulation of cell cycle
Base-excision repair
DSB repair
DSB repair via break induced replication
DSB repair via HR
Log10 P-Value
-Log10 (P-Value)
D.
NT vs IR
-Log2 Fold Change
-Log10 (P-Value)
TFCP2L1
MARCHF4
PLA1A
PPP1R16B
ITGAM
LINC01647
FAM153A
C1orf94
CYP11A1
DAW1
ITGAX
LINC00870
ART3
LOC100996598
SULT2B1
GLDN
E.
dN vs IR
-Log2 Fold Change
-Log10 (P-Value)
RAET1L
MARCHF4
ESM1
MMP1
SOWAHA
CFAP91
F.
IR vs dN +IR (FC>=1.5, p<0.05) 1106 genes
dN + IR
IR
Expression
0.5mM dN +5 Gy IR
5 Gy IR
G.
dN + IR
IR
Expression

Supplemental Figure S2: Transcriptomic analysis reveals distinct gene expression and pathway alterations following dNTP level modulation and IR in GBM cells. A: Boxplots showing the distribution of non-reference mutation counts, as determined by FreeBayes variant calling from RNA-seq data, for control (black) and dN-treated (blue) samples (n = 3 per group). **B:** Volcano plot showing gene expression changes in the dN-only group compared to the control group, highlighting upregulated genes in red and down-regulated genes in blue (P <0.05, fold change >1.5). **C:** Gene ontology enrichment analysis of selected pathways exhibiting significant changes between the dN-only and Control groups. **D/E:** Volcano plot showing gene expression changes in the IR only group (D) and dN + IR group (E) compared to the Control group, highlighting upregulated genes in red and down-regulated genes in blue (P <0.05, fold change >1.5). **F/G:** Differentially Expressed Gene Heatmap (E) and selected heatmap (F) of 5Gy IR only triplicate (red) compared to 5Gy IR + 0.5mM dN treatment (purple).

https://doi.org/10.3390/ijms27188045

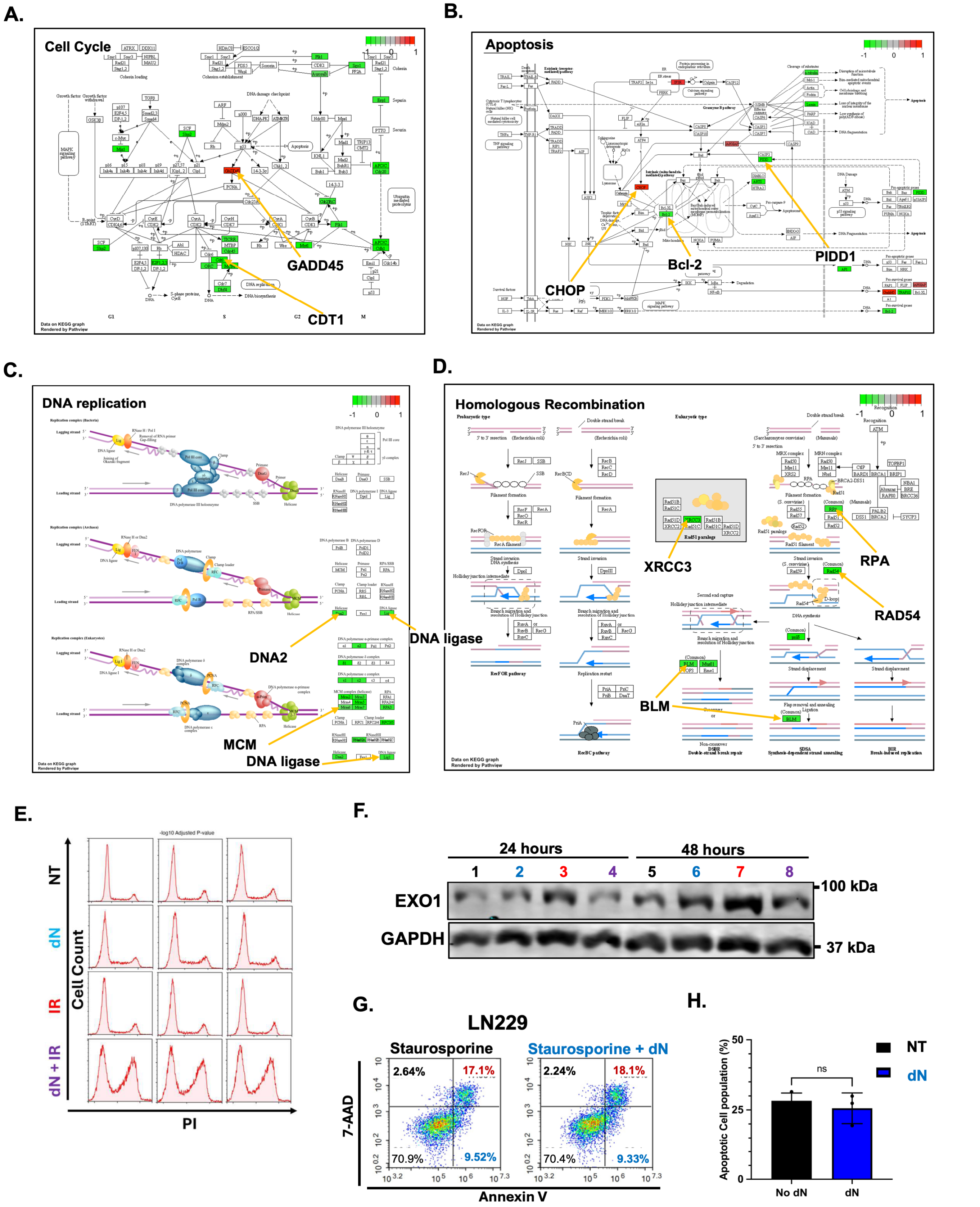

Supplemental Figure S3: Pathway disruption and protein-level validation of HR and cell cycle regulation in LN229 cells treated with dNs and IR. A-D: KEGG Pathway views illustrating differential gene expression in LN229 cells treated with dN + IR compared to those treated with IR alone. Pathways shown include the Cell Cycle (A), Apoptosis

Cascade (B), DNA Replication (C), and Homologous Recombination (D). **E:** Representative flow cytometry histograms showing cell cycle distribution following the indicated treatments. Data were analyzed using the MultiCycle AV module in FCS Express. **F:** Immunoblot of LN229 lysates 24 and 48 hours after the indicated treatment. Lanes 1 and 5= no treatment; lanes 2 and 6= dN alone; lanes 3 and 7= IR alone; and lanes 4 and 8= dN + IR combination treatment. **G:** Annexin V/ 7AAD staining of LN229 cells 48 hours after staurosporine treatment to determine early and late-stage apoptosis. **H:** Quantification of apoptotic LN229 cells (Student's t-test).

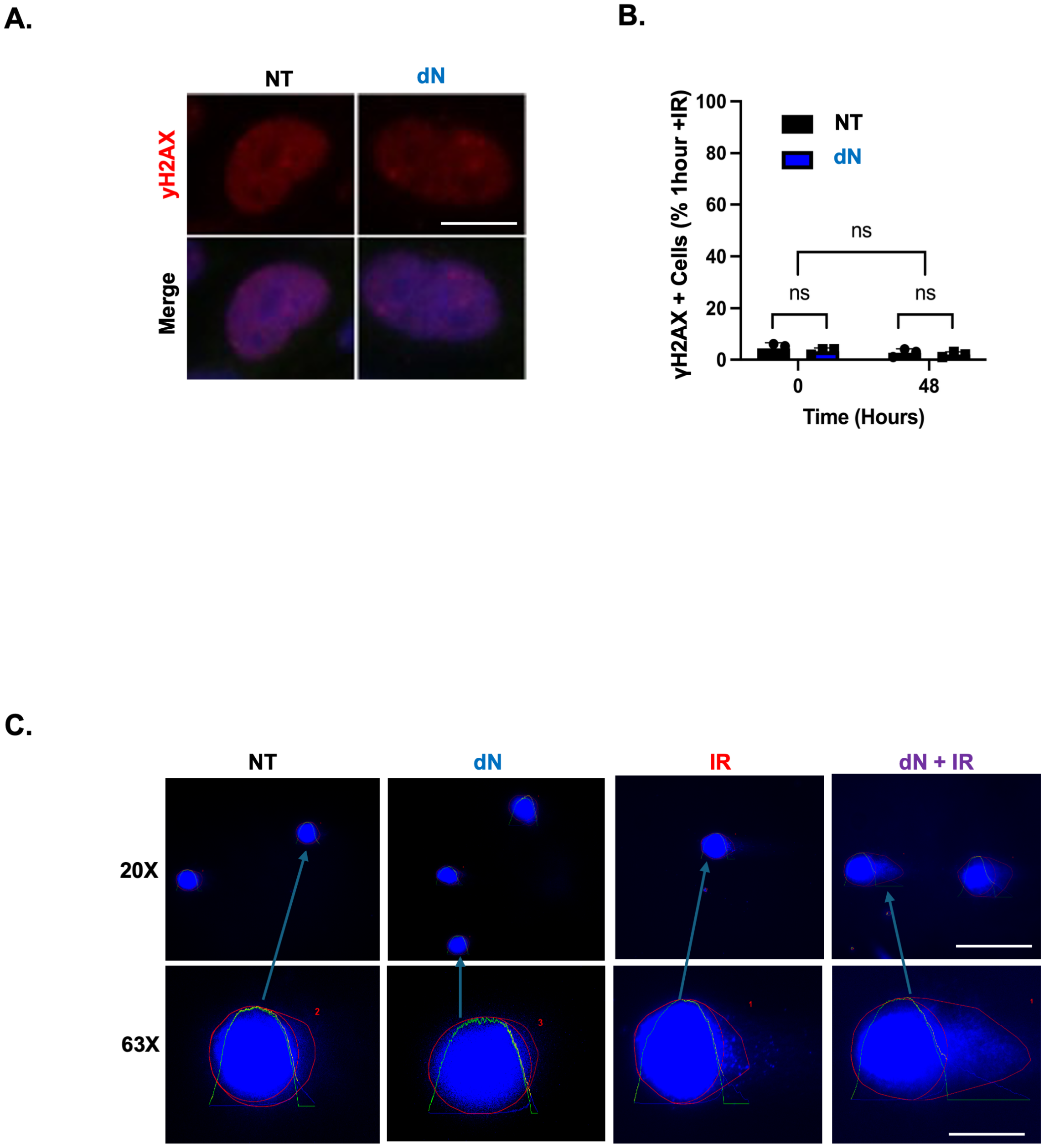

Supplemental Figure S4: Elevated dNTP levels delay the repair of DNA double-strand breaks. A/B: DAPI (blue) and γH2AX (red) merged representative image before or 1 hour after PBS or dN treatment and IR in LN229 with quantification (B) (Two-Way ANOVA). Scale bar, 50 μm. **C:** Neutral comet representative images and quantification from LN229 cells, dN-treated or untreated cells, with and without exposure to 5 Gy IR after 24 hours. Slides were imaged at 63× magnification, and DNA fragmentation was quantified using the OpenComet plugin for Fiji (ImageJ). A minimum

of 50 cells (n = 50) were analyzed for each experimental condition from randomly selected fields. Cells containing >20 discrete foci per nucleus were scored as positive for damage or repair markers. Scale bar, 50 µm (20X) and 10µm (63X).

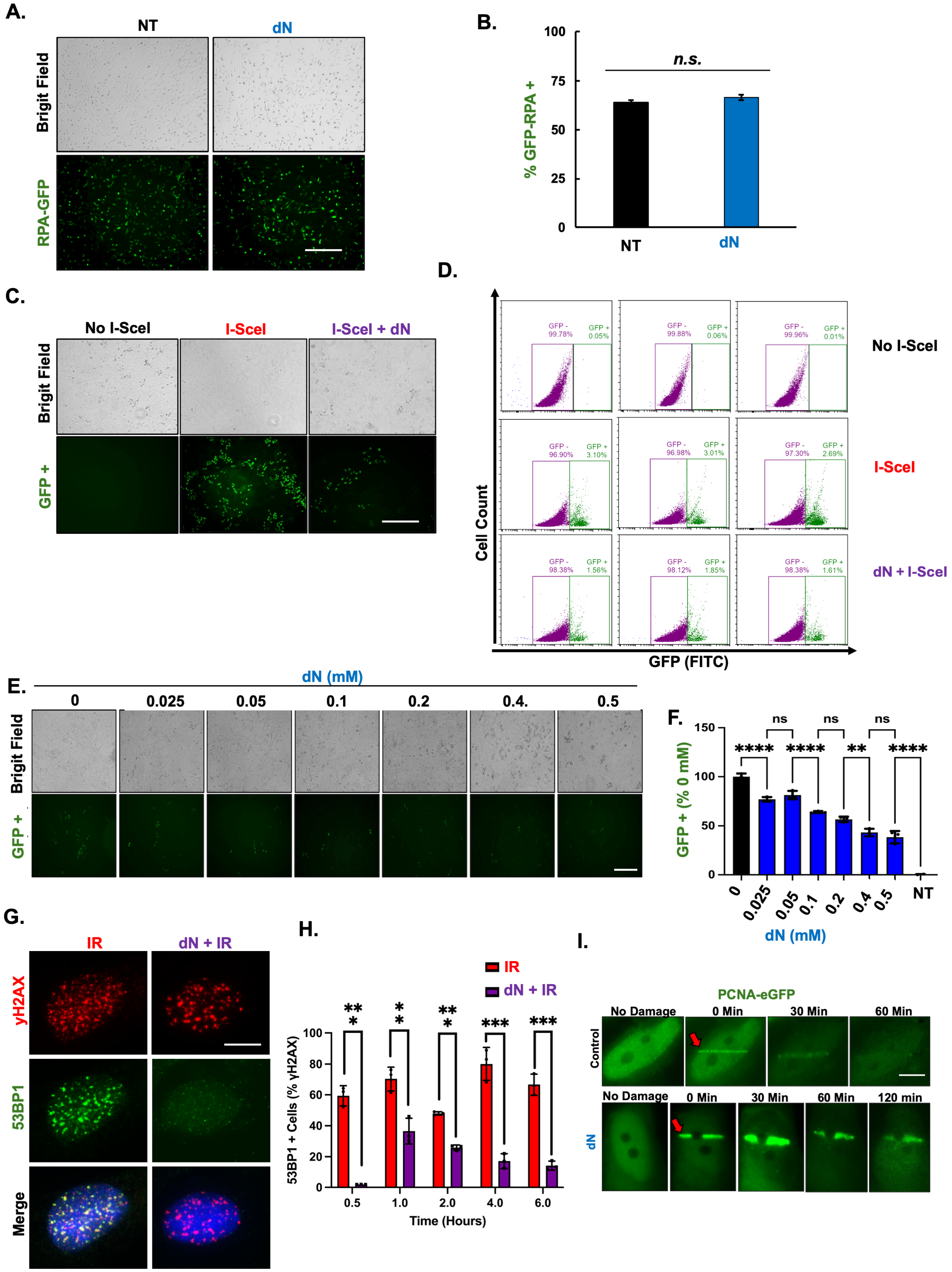

Supplemental Figure S5: dNTP level elevation impairs HR and alters DNA damage response dynamics in GBM cells. **A:** Bright field and GFP representative images of RPA-GFP transfection in cells with no treatment (NT) and 0.5mM dN (dN). Scale bar 400μm **B:** Quantification of RPA-GFP Expression 72 hours after transfection (Student's t-test). **C:** Representative images of cells with integrated HR GFP reporter gene (HR GFP+ cells) treated with no treatment (NT), I-SceI alone (I-SceI), or 0.5mM dN and I-SceI (dN + I-SceI) after 96 hours of I-SceI exposure. **D:** Flow cytometry analysis of HR-mediated DSB repair (GFP+) in HR GFP+ cells treated as described in **C**. **E-F:** HR GFP+ Cells treated with the indicated amount of dN and analysed 96 hours after break induction by I-SceI. **E**: Representative image. Scale bar 400μm **F:** Quantification via flow cytometry (One-Way ANOVA). **G:** γH2AX (red), 53BP1 (green), and DAPI (blue) immunofluorescence staining, representative cell image 6 hours post-5Gy IR treatment with or without dN treatment. Scale bar 10μm **H:** Foci Quantification of γH2AX, 53BP1, and DAPI immunofluorescence staining at 0, 0.5, 1, 2, 4, and 6, hours post-5Gy IR with or without dN treatment. **I:** Representative images from a laser micro-irradiation assay showing PCNA-GFP recruitment over a 2-hour time course. Scale bar 10μm. Cells containing >20 discrete foci per nucleus were scored as positive for damage or repair markers.

https://doi.org/10.3390/ijms27188045

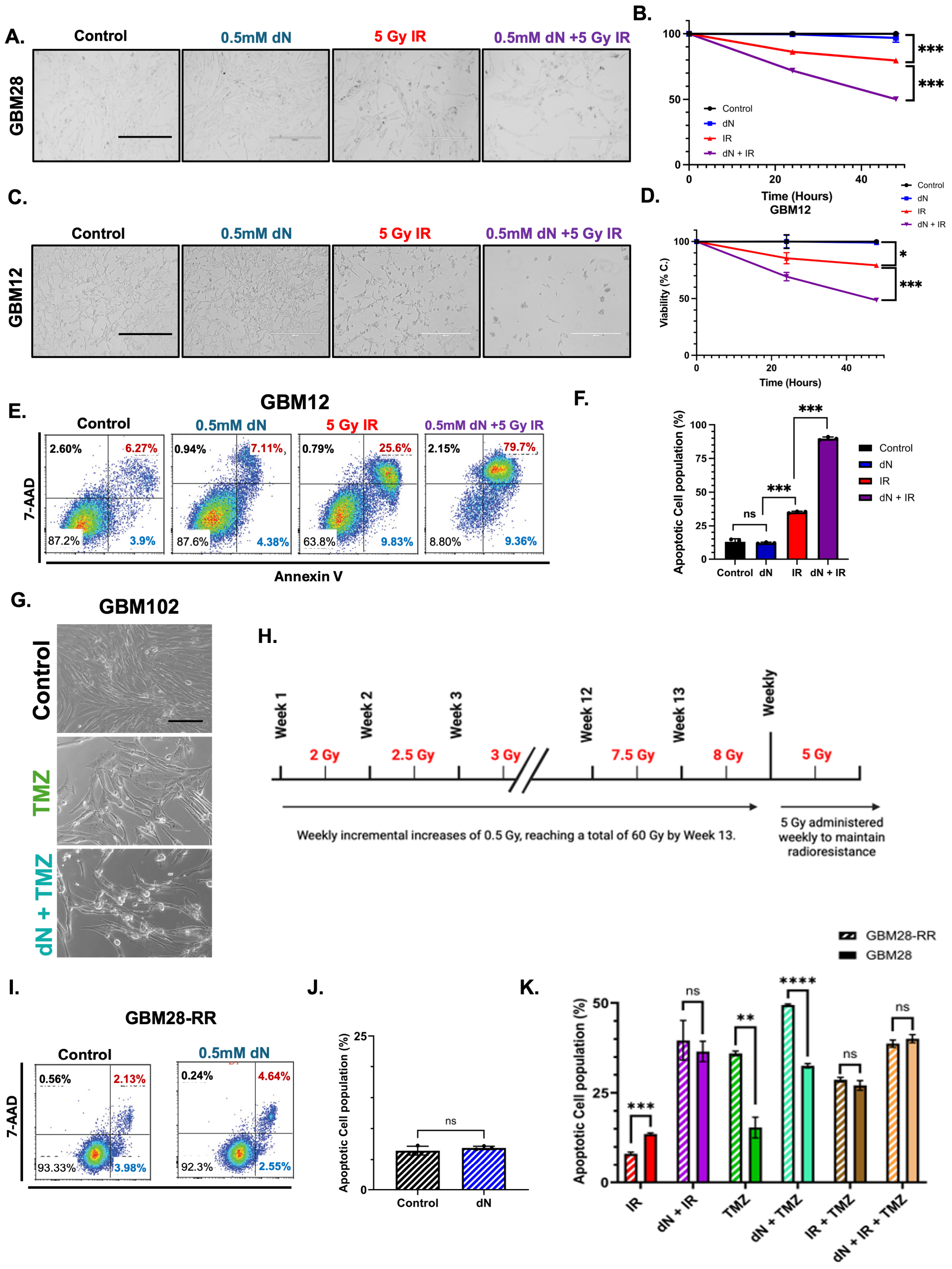

https://doi.org/10.3390/ijms27188045

Supplemental Figure S6: GBM cell viability is reduced under elevated dNTP levels. A: Bright field representative images of GBM28 cells 48 hours after the indicated treatment. Scale bar 400µm. **B:** Cell viability quantified by Alamar Blue staining 48 hours after treatment. **C:** Bright field representative images of GBM12 cells 48 hours after the indicated treatment. Scale bar 400µm**. D:** Cell viability quantified by Alamar Blue staining 48 hours after treatment. **E:** Annexin V/ 7AAD staining of GBM12 cells 48 hours after the indicated treatment to determine early and late-stage apoptosis. **F:** Quantification of apoptotic GBM12 cells. **G:** Brightfield Representative Images of Apoptotic Data in GBM102 48 hours after treatment. Scale bar 100µm **H:** Schematic of radio-resistant cell generation. **I:** Annexin V/7AAD apoptosis assay comparison between control and dN treated GBM28-RR. **J:** Quantification of total apoptotic cell percentage. **K:** Quantification of apoptosis in GBM28 compared to GBM28-RR given the indicated treatment after 48 hours.

Disclaimer/Publisher's Note: The statements, opinions and data contained in all publications are solely those of the individual author(s) and contributor(s) and not of MDPI and/or the editor(s). MDPI and/or the editor(s) disclaim responsibility for any injury to people or property resulting from any ideas, methods, instructions or products referred to in the content.

https://doi.org/10.3390/ijms27188045
